# Effective *in vivo* gene delivery with reduced toxicity, achieved by charge and fatty acid -modified cell penetrating peptide

**DOI:** 10.1038/s41598-017-17316-y

**Published:** 2017-12-06

**Authors:** Kaido Kurrikoff, Kadi-Liis Veiman, Kadri Künnapuu, Elin Madli Peets, Tõnis Lehto, Ly Pärnaste, Piret Arukuusk, Ülo Langel

**Affiliations:** 1 Institute of Technology, University of Tartu, Nooruse 1, 50411 Tartu, Estonia; 2 Department of Neurochemistry, The Arrhenius Laboratories for Natural Sciences, Stockholm University, SE-10691 Stockholm, Sweden; 3 Present Address: Institute of Technology, University of Tartu, Tartu, 50411 Estonia

## Abstract

Non-viral gene delivery systems have gained considerable attention as a promising alternative to viral delivery to treat diseases associated with aberrant gene expression. However, regardless of extensive research, only a little is known about the parameters that underline *in vivo* use of the nanoparticle-based delivery vectors. The modest efficacy and low safety of non-viral delivery are the two central issues that need to be addressed. We have previously characterized an efficient cell penetrating peptide, PF14, for *in vivo* applications. In the current work, we first develop an optimized formulation of PF14/pDNA nanocomplexes, which allows removal of the side-effects without compromising the bioefficacy *in vivo*. Secondly, based on the physicochemical complex formation studies and biological efficacy assessments, we develop a series of PF14 modifications with altered charge and fatty acid content. We show that with an optimal combination of overall charge and hydrophobicity in the peptide backbone, *in vivo* gene delivery can be augmented. Further combined with the safe formulation, systemic gene delivery lacking any side effects can be achieved.

## Introduction

Gene therapy has a potential to treat variety of diseases which result from aberrant gene expression in cells and tissues. Administration of free nucleic acids (such as plasmid DNA, pDNA) without vectorization is not efficient and therefore, delivery vectors are needed. To make pDNA resistant to nuclease attack and available for cellular uptake, vectorization with a delivery system and nanoparticle formation are often performed. In order to develop delivery vectors that have clinical potential, nucleic acid cargo should be transported via systemic administration (so-called *in vivo* transfection). However, *in vivo* transfection poses a significant challenge, related to insufficient delivery and safety concerns^1^.

Cell penetrating peptides (CPPs) are a class of promising non-viral delivery vectors for the delivery of nucleic acid biomolecules^1,2^. However, they share several common drawbacks with other known cationic polymer –based delivery systems. Firstly, when systemically administered, the CPP–nucleic acid complexes may become unstable and are rapidly opsonized, degraded, and/or dissociated^3^. On the other hand, if they are too inert to form any contacts with cell membranes, they will be eliminated without having achieved any biological effect^4^. Secondly, once reaching the cell membrane, they should be able to be both taken up by the cells and be efficiently delivered into the nucleus, thereby passing several critical biological barriers^3,5^. Solving these drawbacks in a safe and efficient way should be part of the comprehensive optimization of the delivery platform.

We have previously shown that a CPP, PF14^6^ is an efficient plasmid DNA delivery vector in both cell cultures^7^ and in mouse models after systemic administration^4^. PF14 is similar to cationic liposomes and PEI-based polymers^8,9^, however, this treatment gave rise to side-effects such as occasional acute toxicity. Previously we improved the *in vivo* delivery by using PEGylation, which allowed inhibiting unwanted interactions with blood components^4^, effectively eliminating the side effects. Unfortunately, this strategy is only applicable for specific diseases, such as for the tumor-targeting, and requires delicate optimization, which is characterized by the phrase “PEG dilemma”^10^. The present study aims to address the two abovementioned major issues with *in vivo* transfection in a way that does not include inhibition of transfection with a biological shield, yet is able to eliminate the side effects, using a cell penetrating peptide PF14 as an example. In order to achieve this, we introduce the CPP/pDNA nanoparticles to a series of challenges that resemble specific barriers of *in vivo* delivery. Based on the performance in these challenging environments, we are able to propose a modified PF14 peptide with optimal amphipathicity tailored for the *in vivo* pDNA delivery that offers more efficient transfection with no side effects.

## Materials and Methods

### Synthesis of peptides

The sequence information of all the peptides used in the current study are presented in Table 1.

**Table 1:** The peptide sequences used in the current study.

**Name**	**PF iteration**	**Sequence**	**Modifications/Properties**
PF14		CH_3_(CH_2_)_16_-CONH-AGYLLGKLLOOLAAAALOOLL-NH_2_	Net charge +5
PF6		CH_3_(CH_2_)_16_-CONH-AGYLLGK^(a)^INLKALAALAKKILL-NH_2_	Net charge +10^(a)^
**PF14 net charge modifications**			
PF14-O	PF1450	CH_3_(CH_2_)_16_-CONH-AGYLLGKLLOOLA**OO**ALOOLL-NH_2_	A14 −>O, A15 −>O Net charge +7
PF14-E	PF1451	CH_3_(CH_2_)_16_-CONH-AGYLLGKLL**E**OLAAAALOOLL-NH_2_	O10 −>E Net charge +3
**PF14 fatty acid chain modifications**			
C0-PF14	PF150	NH_2_-AGYLLGKLLOOLAAAALOOLL-NH_2_	No fatty acid Net charge +6
C10-PF14	PF155	CH_3_(CH_2_)_8_-CONH-AGYLLGKLLOOLAAAALOOLL-NH_2_	Carbon tail length 10 Net charge +5
C22-PF14	PF161	CH_3_(CH_2_)_20_-CONH-AGYLLGKLLOOLAAAALOOLL-NH_2_	Carbon tail length 22 Net charge +5
**PF14 net charge and fatty acid chain modifications**			
C22-PF14-O	PF1452	CH_3_(CH_2_)_20_-CONH-AGYLLGKLLOOLA**OO**ALOOLL-NH_2_	A14 −>O, A15 −>O Carbon tail length 22 Net charge +7

^(a)^Four trifluoromethylquinoline moieties attached to succinylated lysine tree; overall calculated charge of the peptide is +10 at pH 7.4. O=ornithine.

All peptides were synthesized in a stepwise manner in a 0.1 mmol scale on an automated peptide synthesizer (Initiator Alstra, Biotage) by using standard protocols for Fmoc solid-phase synthesis. Rink-amide – ChemMatrix resin (Biotage) was used as the solid phase to obtain C-terminally amidated peptides. N-terminal fatty acid residue was coupled by treatment of peptidyl resin with 4 equiv of fatty acid (Sigma-Aldrich), 4 equiv of HOBt/HBTU (Multisyntech), and 8 equiv of DIEA (Sigma-Aldrich) in DMF/DCM for 18 h.

PF6 was synthesized as described earlier^11^. Briefly, Mtt protecting group from Lys7 was removed by repeated washes with 0.1%TFA and 3%TIS in DCM. Fmoc-Lys (Fmoc) (Iris Biotech) was coupled two times to create a lysine-tree to which firstly succinic anhydride (Sigma-Aldrich) and thereafter trifluoromethyl quinoline derivative were coupled.

The final cleavage was performed using a standard protocol [95% trifluoroacetic acid (TFA)/2.5% TIS/2.5% water] for 2 h at room temperature. Peptides were purified by RP-HPLC using a C3 column and 5–80% acetonitrile (0.1% TFA) gradient. Molecular weight of the peptides was analyzed by MALDI-TOF mass-spectrometry (Brucker), and purities were >90% as determined by analytical HPLC.

### Complex formation

If not indicated otherwise, complexes were formed using 0.5 μg of pDNA (pGL3, Promega, Sweden) mixed with a peptide at indicated charge ratio (N/P, nitrogen-to-phosphate ratio) in MQ water in a final volume of 50 μl. N/P was calculated theoretically and accounted for both the positive charges of the peptide (Table 1) and negative charges of the pDNA. Thereafter, complexes were incubated for 40 minutes at room temperature prior to each experiment.

For *in vivo* studies, 20–50 µg of p-CMV-Luc2 (marked as pLuc in the text) per animal was mixed with a peptide at indicated N/P in MQ in a final volume of 100 µl. After 40 minutes of incubation, glucose was added to a final 5% glucose in 200 μl and immediately injected intravenously via the tail vein.

jetPEI was mixed according to the protocol provided by the manufacturer. Briefly, 20 µg of pLuc was diluted in 50 µl of MQ. In a separate test tube, jetPEI (N/P8) was diluted in MQ in a final volume of 50 µl. Thereafter, 50 µl of 10% glucose was added to both solutions and jetPEI solution was added to the pDNA solution, and incubated at room temperature for 40 minutes. For *in vitro* experiments, jetPEI/pDNA was formulated as described above except that the resulting complexes were subsequently diluted to the required pDNA dose using a 5% glucose solution.

### Gel shift and EtBr exclusion assay

pDNA condensation and incorporation into complexes was analyzed using a gel shift assay and an ethidium bromide (EtBr) (Sigma, Sweden) exclusion assay. Briefly, for both assays, complexes were formed at N/P4. For the gel shift assay, 10 µl of complexes (0.01 µg/µl pDNA; N/P4) were mixed with 2 µl DNA loading dye and thereafter electrophoresed on agarose gel (1%) in TAE (1X) and visualized by staining the gel with EtBr (0.5 μg/ml) and imaged under UV light.

In the EtBr exclusion assay, after 40 minutes of incubation, 165 μl of MQ water was added to each complex sample (20 µl; 0.01 µg/µl pDNA; N/P4) and transferred into a black 96-well plate (NUNC, Sweden). Thereafter, 15 μl (5.33 μg/ml) of EtBr solution was added to give a final EtBr concentration of 400 nM. After 15 minutes, the fluorescence was measured on a Spectra Max Gemini XS fluorometer (Molecular Devices, Palo Alto, CA) at λ_ex_ = 518 nm and λ_em_ = 605 nm. The results are expressed as relative fluorescence and a value of 100% is attributed to the fluorescence of uncomplexed pDNA with EtBr.

### Resistance against enzymatic degradation

For the evaluation of complex stability in FBS, gel shift assay was performed, as follows: 50% of FBS was added to the pre-formed complexes (N/P2 for the peptides, N/P8 for jetPEI) and incubated at 37 °C. Samples were collected from the tube with the complex+FBS after the time points of 0.5, 1, 3, 6, 24 and 48 h, and transferred to a new tube for a further treatment with heparin sodium salt + Triton X-100 (final concentrations 3.33 mg/ml and 0.1%, respectively) (or an equal volume of MQ) and incubated for further 10 min at room temperature. Then the samples were mixed with the DNA loading dye and electrophoresed exactly as stated above in the Gel shift assay description, using 0.1 µg of pDNA per lane. ZipRuler Express DNA Ladder 2 (ThermoFisher Scientific) was added to each gel.

The effect of degradative enzymes to the stability of preformed CPP/pDNA complexes was evaluated using DNase I and proteinase K (Thermo Scientific, USA). In the nuclease stability assay, DNase was added to the complexes (1 µg pDNA at N/P2 and N/P4 in 100 µl; DNase final concentration was 0.02 U/µl) and incubated for 30 minutes at 37 °C. The nondigested control samples received an equal amount of DNase buffer (without the enzyme). Thereafter, the complexes were dissociated by adding 5 µl of heparin (20 mg/ml) and 5 µl of 0.6% Triton X-100 solution in PBS and further incubated for 10 minutes at room temperature. Following this, the remaining pDNA amount was quantified using EtBr.

For the proteinase K treatment, 30 μl of pre-formed CPP/pDNA complex solution (0.3, 0.2, 0.1 and 0.05 µg of pDNA per sample) or CPP in MQ was transferred to a black 96-well plate and 90 µl of MQ was added. After a short period of mixing, fluorescent DNA intercalating dye dilution was added (Quant-iT™ PicoGreen®, ThermoFisher Scientific) and incubated for 5 minutes at room temperature. The fluorescence was measured (Synergy MX, BioTek; λ_ex_ 492 and λ_em_ 535) to quantify the initial accessibility of pDNA. Thereafter 10 μl of Proteinase K (Thermo Scientific) was added with the final enzyme concentration ~2.4 U per well. Fluorescence was measured over an indicated period at 26 °C. The readings were normalized against the fluorescence of free pDNA.

### Dynamic light scattering (DLS) measurements

The mean hydrodynamic diameter of the nanoparticles was determined by analyzing the dynamic light scattering using a Zetasizer Nano ZS (Malvern Instruments, United Kingdom). CPP/pDNA complexes were prepared as described above, transferred to a disposable low volume cuvettes and the size of the complexes was measured over a period of time at room temperature. For studying the effects of serum incubation, an equivolume amount of FBS (Sigma) solution in MQ was added to the complexes before transferring to the cuvettes. Each sample was prepared in duplicates and was measured in 3 technical replicates (10 runs, 1 run = 10 seconds) were made.

To measure the zeta potential, the complexes were prepared in MQ in a final volume of 300 µl with 0.01 µg/µl of pDNA. After 40 minutes of incubation at room temperature, the complexes were diluted in MQ to a final volume of 1 ml. For measuring serum effects, 300 µl of FBS solution in MQ was added to the complexes, followed by dilution to a final volume of 1 ml with MQ. The measurements were performed over a period of time at room temperature. Each sample was prepared in duplicates and was measured in 3 technical replicates; 1 measurement was set to 10 runs.

### Assessment of opsonization with SDS-PAGE

The complexes were formed as described above and incubated with equal volume of FBS for 40 min at room temperature. The complexes were then centrifuged for 90 min at 18,625 × g. The formed pellet was washed 5 times with MQ. The samples were then treated with SDS and subjected to PAGE (NuPAGE™ 4–12% Bis-Tris Protein Gel, Invitrogen). The protein was visualized with coomassie (ThermoFisher Scientific SimplyBlue™ SafeStain). The gels were imaged and protein lanes were quantified with ImageJ (https://imagej.nih.gov/ij/).

### Cell culture transfection

All cell lines were grown in a humidified environment at 37 °C with 5% CO_2_. CHO cells were cultivated in Ham’s F12 media, supplemented with 0.1 mM non-essential amino acids, 1.0 mM sodium pyruvate, 10% FBS, 100 U/ml penicillin and 100 µg/ml streptomycin (PAA Laboratories GmbH, Germany).

For the plasmid delivery assay, 4 × 10^4^ CHO cells were seeded 24 hours prior to each experiment in 24-well plates. Cells were treated with CPP/pDNA complexes at indicated N/P for 4 h in serum containing media, followed by the addition of 1 ml 10% serum-containing medium and incubated for another 20 hours. Thereafter, cells were washed with 1x PBS and lysed using 100 µl 0.1% Triton X-100 in PBS for 15 min at 4 °C. Luciferase activity was measured using Promega’s luciferase assay system according to the manufacturer’s protocol using a GLOMAX™ 96 microplate luminometer (Promega, Sweden) and normalized to the protein content (BioRad Protein Assay, USA).

### Activity towards cell membranes

The ability of the peptides to lyse the red blood cells (RBC) in PBS was used as a measure of membrane activity. To obtain fresh mouse RBC, blood was collected from the saphenous vein using sodium-heparinized (80 iu/ml ± 30%) blood collection capillaries (Marienfeld, Germany) and transferred to heparinized blood collecting tubes (Sarstedt, Germany). To isolate RBCs, the whole blood was centrifuged for 15 minutes at 300 × g, followed by removal of the plasma. The RBCs were then washed three times with 1 × PBS (RBC pelleting with 15 minutes at 300 × g). The resulting RBCs were then resuspended in PBS at 4% (v/v).

For the analysis, 200 μl of RBC suspension was added to 200 μl of peptide solution or peptide/pDNA complexes in 5% glucose (final RBC concentration 2%) and gently vortexed. The peptide/pDNA complexes were prepared using a fixed 1 μg of pDNA and varying amount of the peptide. Following this, samples were incubated for 1 hour at 37 °C with gentle stirring. After incubation, the RBCs were pelleted (15 minutes at 300 × g) and the amount of hemoglobin in the supernatant was assessed by measuring the absorbance at 540 nm using a 96-well microplate reader. The experiments were conducted in triplicates.

100% of hemolysis was defined as the RBCs treated with 0.1% Triton-X100 in PBS and 0% was obtained with cells incubated in 5% glucose solution. % of hemolysis was calculated by the formula: % Hemolysis = [(A_sample_ − A_glucose_)/A_triton_ − A_glucose_)] × 100.

### Quantitation of luciferase activity from tissues

All animal experiments and procedures were conducted according to the approval by the Estonian Laboratory Animal Ethics Committee (approvals no. 69 and 70, dated Feb 9, 2011 and no 81 from Apr 04, 2016); all the experiments were performed in accordance with relevant guidelines and regulations.

Reporter gene expression levels were evaluated post mortem 24 h after injecting the complexes into the tail vein, using male and female BALB/c mice (8-week-old). Each animal received a single 200 μl i.v. injection of the complexes. The mice were sacrificed using cervical dislocation and tissues were harvested and snap-frozen on dry ice. The whole tissues were homogenized using a Precellys®24-Dual homogenization system (Bertin Technologies, France) and lysed using 1 × Promega lysis buffer (Promega, Sweden). The content of luciferase was analyzed as described previously^12^. Briefly, homogenized tissues were thawed and 500 µl of Promega Reporter lysis 1 × buffer was added. The samples were subsequently vortexed for 15 minutes, subjected to three consecutive freeze-thaw cycles (liquid nitrogen and 37 °C water bath), centrifuged for three minutes at 10,000 x g, 4 °C; the supernatant was removed and saved for later analysis. 500 µl of lysis buffer was again added to the pellet and the extraction process repeated (without freeze-thaw cycles). The second supernatant was combined with the first one and subjected to luciferase activity assay. Luciferase was measured with Promega luciferase assay system, in combination with GLOMAX 96 microplate luminometer (Promega, Sweden). For this, 20 µl of the supernatant was transferred to the white 96-well plate and 80 µl of luciferase substrate was added to each sample. An average LU (light unit) out of three technical replicates was used for data analysis. The average LU values within each sample were normalized to the protein content (BioRad, United States) and the resulting RLU/mg were further normalized to the corresponding tissue of the animals that received the naked pDNA injection (sham treatment).

### Histological analysis

The physical accumulation of the complexes were assessed by using Cy5-labeled pDNA (Mirus, USA). Equal amounts of labelled pDNA were administered, regardless of the N/P ratio of the complexes (e.g. N/P4 included 20 μg of labelled pDNA, whereas in case of N/P2, 20 μg of labelled pDNA was mixed with 30 μg of unlabeled pDNA). For the histological evaluation, the lungs were inflated postmortem with 1:1 cryomatrix:PBS (O.C.T embedding matrix, Kaltek, Italy), frozen in 2-methylbutane bath on dry ice. Fresh cryo-sections (10 μm thick) were fixed with ice cold methanol, counterstained with DAPI (AppliChem) and imaged with confocal microscopy (Zeiss LSM710).

The data are presented as the mean +− standard deviation.

## Results and Discussion

### Optimization of the nanoparticle formulation for *in vivo* pDNA delivery reduces side effects

**Figure 1:**
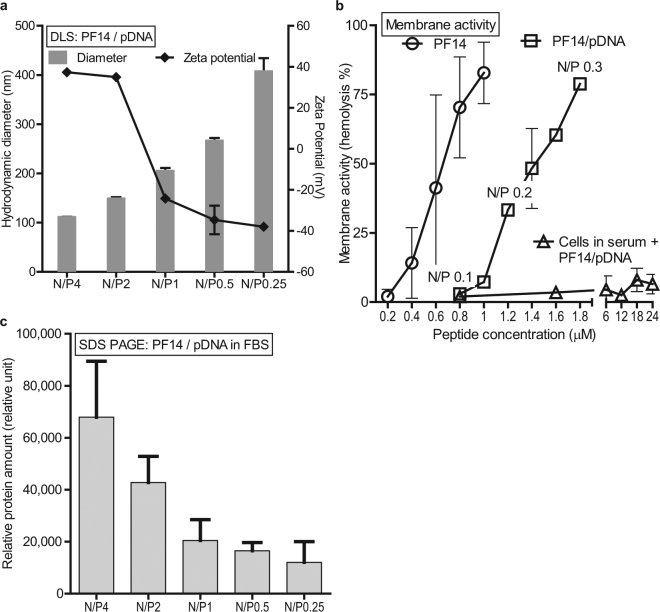
Reduction of N/P of PF14/pDNA allows neutralization of the surface charge and reduction of opsonization of the particles. (**a**) The average size and zeta potential of PF14/pDNA complexes at decreasing N/P was evaluated using DLS. (**b**) Membrane disturbing activities of PF14 peptide and PF14/pDNA complexes are assessed through the hemolysis assay, where the liberation of hemoglobin from mouse RBCs is used as a measure of “membrane activity”. Data is represented as hemolysis %, where 100% represents hemolysis of all cells (Triton X-100) and 0% represents treatment with a neutral solution (5% glucose). (**c**) Opsonization of the PF14/pDNA nanoparticles with FBS. The nanoparticles were incubated in serum, separated, and quantitated after SDS-PAGE. The nanoparticle-associated total protein amount is expressed in arbitrary units. The values are the means of two experiments.

The size and surface charge are among the crucial parameters that affect the fate and activity of nanoparticles *in vivo*. For example, particle size affects the clearance and passive accumulation to tumors^3^. It has been argued that a size of more than 10 (to avoid first pass elimination by the kidney) and less than 150 nm (to be able to extravasate and diffuse in the interstitium) is optimal from the delivery point of view^13^. The particle surface charge affects binding to blood plasma proteins, resultant opsonization^14^, and thus affects both efficacy (through elimination from the organism) and side effects (activation of immune system). We have previously developed PF14, a CPP that efficiently transfects pDNA in cell cultures^7^ and *in vivo* ^4^. PF14 forms nanoparticles with pDNA that are well within the suggested range, but display rather high cationic zeta potential (Fig. 1) at N/P4, as was used in these studies. Reduced surface charge and opsonization is most commonly achieved through PEGylation, which acts as an inert hydrophilic barrier between the particle and the extracellular environment. Indeed, we have previously utilized the same strategy, reporting *in vivo* optimized, PEGylated PF14 transfection system^4^. However, the disadvantage of PEGylation is that it also reduces favorable contacts and transfection efficacy and thus, additional mechanism for eliminating PEG has to be incorporated into the nanoparticle^4^.

In the current report, we assessed the characteristics of the nanocomplexes in various challenging environments that mimic several aspects of *in vivo* conditions. We aimed to retain the simplicity of the formulation composition (only 2 components needed) and therefore tried to modify (i) the formulation and (ii) the peptide component in the complex to gain maximal pDNA delivery.

We started by titrating the PF14/pDNA N/P ratio, plotting the hydrodynamic sizes and zeta potential of the particles. Figure 1a shows that the decrease in N/P is accompanied with increase in particle size, but also reduction of the zeta potential. Indeed, the surface charge can be easily reduced by lowering the N/P ratio, whereas near-neutral particles are formed at N/P1 (Fig. 1a). The stepwise increase in particle size is probably indicative of (i) reduced repulsion of more charge-neutral particles and (ii) looser interactions between the increased number of nucleic acid molecules and the CPP. As stated above, small particle size and near-neutral zeta would be desirable from the delivery point of view. Therefore, we hypothesized that particles with reduced surface charge are less attractive to opsonins in blood serum and are more efficient *in vivo*.

To have an understanding of the relationship between the surface charge and bioactivity, we gauged the activity of the peptide and complexes towards cellular membranes. For that we used a simple model for assessing cell membrane disturbance, utilizing red blood cells (RBC), separated from their natural environment (the serum) and assessed the liberation of hemoglobin as a response to incubation with a CPP. As expected, the membrane activity increases with peptide concentration (Fig. 1b). When complexed with nucleic acid, the membrane activity of the peptide was reduced for about 2-fold, although the concentration dependence was retained. However, the membrane activity was practically abolished when the same was performed in the presence of serum. Therefore, this assay does not directly reflect the events that occur in the blood stream, but represents a measure for a general cell membrane disturbing activity that may be needed e.g. for the endosomal escape.

We assessed if the opsonization of PF14 complexes could be avoided by lowering the N/P^14^. For that, serum proteins bound to CPP/pDNA complexes were quantified using SDS-PAGE. Indeed, lowering the N/P from 4 to N/P2 reduced the opsonization by approximately 1/3, and further transition to N/P1 reduced the amount of bound protein by another 1/3 (Fig. 1c). It is interesting that although the DLS demonstrated similar zeta potential for both N/P4 and N/P2 (Fig. 1a), less protein was bound with the lower ratio complexes. These results suggest that complexes at high N/P form particles with desired size range of 100 nm, display high activity towards the cell membranes, but are also efficiently opsonized. With the lower N/P, on the other hand, the particle size gradually increases, but less serum proteins are attracted—a feature that may be beneficial when administering the particles i.v.

**Figure 2:**
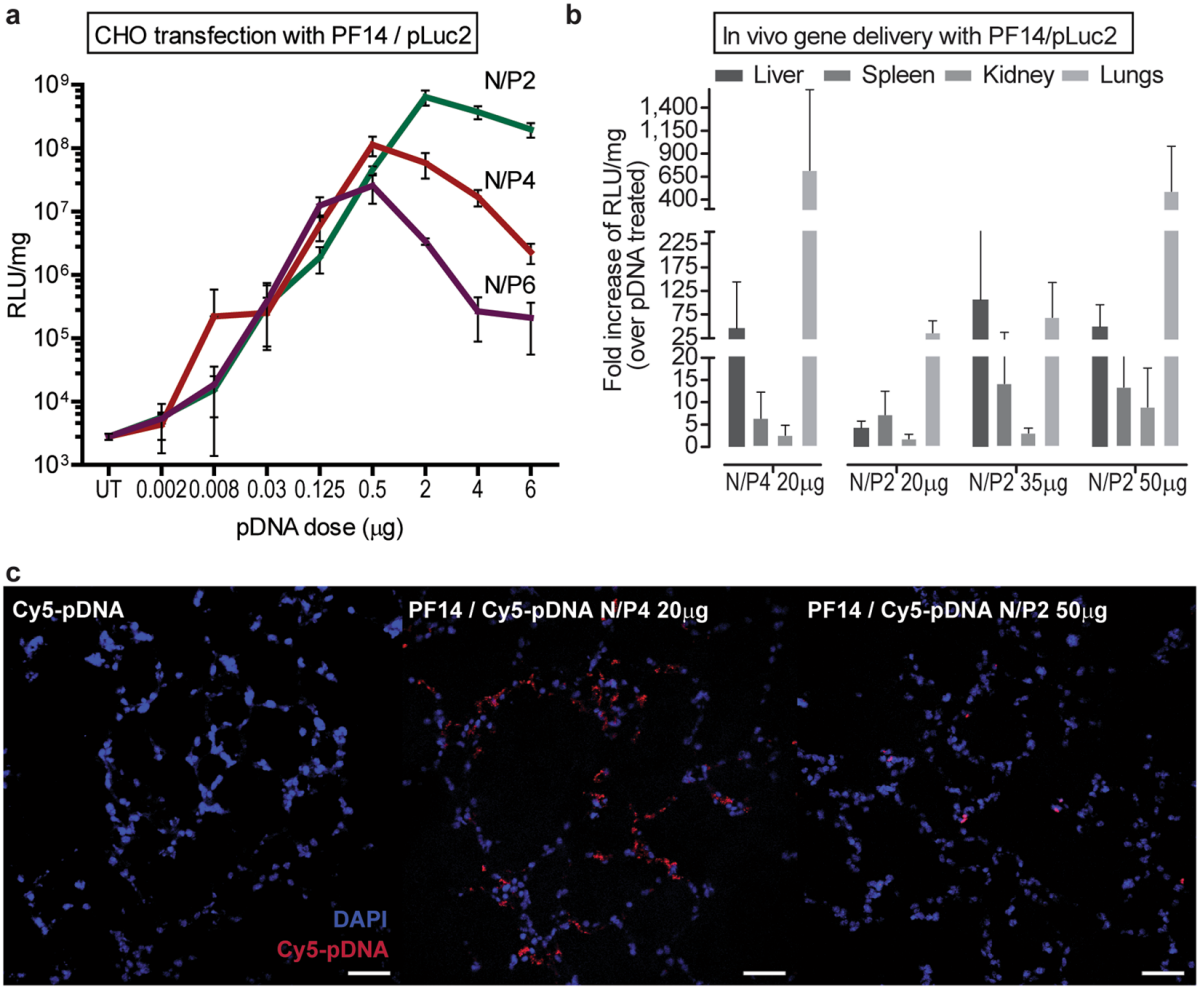
Transfection toxicity can be reduced and efficacy retained by a combination of lowering the N/P and increasing the dose. (**a**) The effect of increasing pLuc dose (up to 6 μg) on transfection efficacy in CHO cells using three different N/P ratios for complexing PF14 with pDNA. (**b**) *In vivo* reporter gene delivery efficacies of CPP/pLuc at N/P4, compared to N/P2. pDNA doses, formulated with PF14 at indicated N/P are shown in the X axis. Data are represented as a fold increase of RLU/mg over sham treatment (using the same dose of naked pDNA). (**c**) Accumulation of PF14/pDNA nanoparticles in lung tissue, using Cy5-labelled pDNA. The cryosections of the lungs represent 1 h post-injection. Scale bar 50 μm.

We therefore asked the question—do the nanoparticles behave differently dependent on the N/P? We were specifically interested in two types of behaviors: (i) the efficacy of transfection and (ii) the toxicity towards the cells. Titrating the pDNA dose showed that in general, the transfection efficacies were not N/P dependent, at least with the PF14 reagent, and we could see increase of transfection with increased pDNA dose, regardless of the used N/P (Fig. 2a). However, increasing the dose beyond the typical (0.5 μg pDNA in 24-well plate) led to increase of the number of dead cells and the change of morphology showed further signs of toxicity, ultimately reflecting in the reduction in transfection. Interestingly, the onset of the toxic effects were clearly dependent on the N/P: at N/P4, the maximum transfection was achieved with the (standard) dose of 0.5 μg, while N/P2 formulation allowed the use of much higher dose (up to 2 μg) (Fig. 2a). On the other hand, increase of the N/P to 6 reduced the usable dose to 0.125 μg. Such a dependence suggests that lowering the N/P allows the use of higher doses, reducing toxicity and increasing efficacy and we were interested if such relationship could also be seen *in vivo*.

Systemic *in vivo* activity of PF14 has been shown before, where efficient transfection occurred in the lungs, but due to the occasional toxic effects there is a need for improvement^4^. Inspired by the cell transfection studies, we lowered the N/P from 4 to 2 and importantly, observed no side effects (zero lethality with N/P2) throughout all the experiments presented hereafter. Unfortunately, simply lowering the N/P from 4 to 2 led to the reduction of transfection efficacy (Fig. 2b, N/P4 20 μg vs N/P2 20 μg, Tukey *post hoc* p < 0.01 after significant ANOVA F_(9,72)_ = 2.02, p < 0.05). Again, based on the cell transfection studies, we then increased the pDNA dose, using the safer N/P2. Indeed, with 50 μg of pDNA, similar efficient gene expression was achieved as with 20 μg at N/P4 (Fig. 2b, N/P4 20 μg vs N/P2 50 μg not significant Tukey p > 0.99). Because the lungs are a critical organ from both the bioactivity and side effects perspective^8,9^, we assessed the accumulation of the complexes in lungs, following the i.v. administration of complexes with fluorescently labeled pDNA. As expected, considerable amount of the fluorescence was observed in lungs after injection with the N/P4 complexes (Fig. 2c), whereas significantly lower signal was present in case of N/P2. This also demonstrates that physical accumulation of the nanoparticles does not necessarily reflect in the transfection, because the N/P2 particles mediate similar gene expression in lungs as with N/P4, but show much less accumulation. We have previously reported reduced accumulation in lungs and reduced side effects using PEGylated peptide for formulation of the complexes^4^. The formulations described here could be a simple and effective alternative to PEGylation.

### Increasing the charge density of the CPP increases gene transfection

It is important to develop delivery vectors that have less side effects, because safety concerns have been reported for all nanoparticle delivery vectors, including PEI^9^ and cationic liposomes^8^. We were interested if the adverse reactions could be reduced by decreasing the amount of the CPP in the transfection mixture. Because the delivery vector is mixed with DNA according to the N/P ratio, one way to reduce the amount of the CPP is by increasing its net charge. We developed PF14 analogue that had a net charge increase by +2 (PF14-O, where two alanines are substituted with ornithines, (Table 1) and compared the transfection efficacy with an analogue that had its net charge decrease by +2 (PF14-E, ornithine to glutamic acid change). In addition, PF6, a well-established peptide for siRNA delivery^11^, that has a net charge of +10 at pH 7.4^15^ was also used. Finally, polyethylenimine (PEI) is a commonly used transfection vector^16^ that exhibits very high charge density, especially when compared to the CPPs listed in Table 1, but similarly to PF14, it efficiently condenses and transfects pDNA and mediates transgene expression in lungs^17^. Therefore, a commercial PEI-based reagent *in vivo* jetPEI was also included for the comparison.

**Figure 3:**
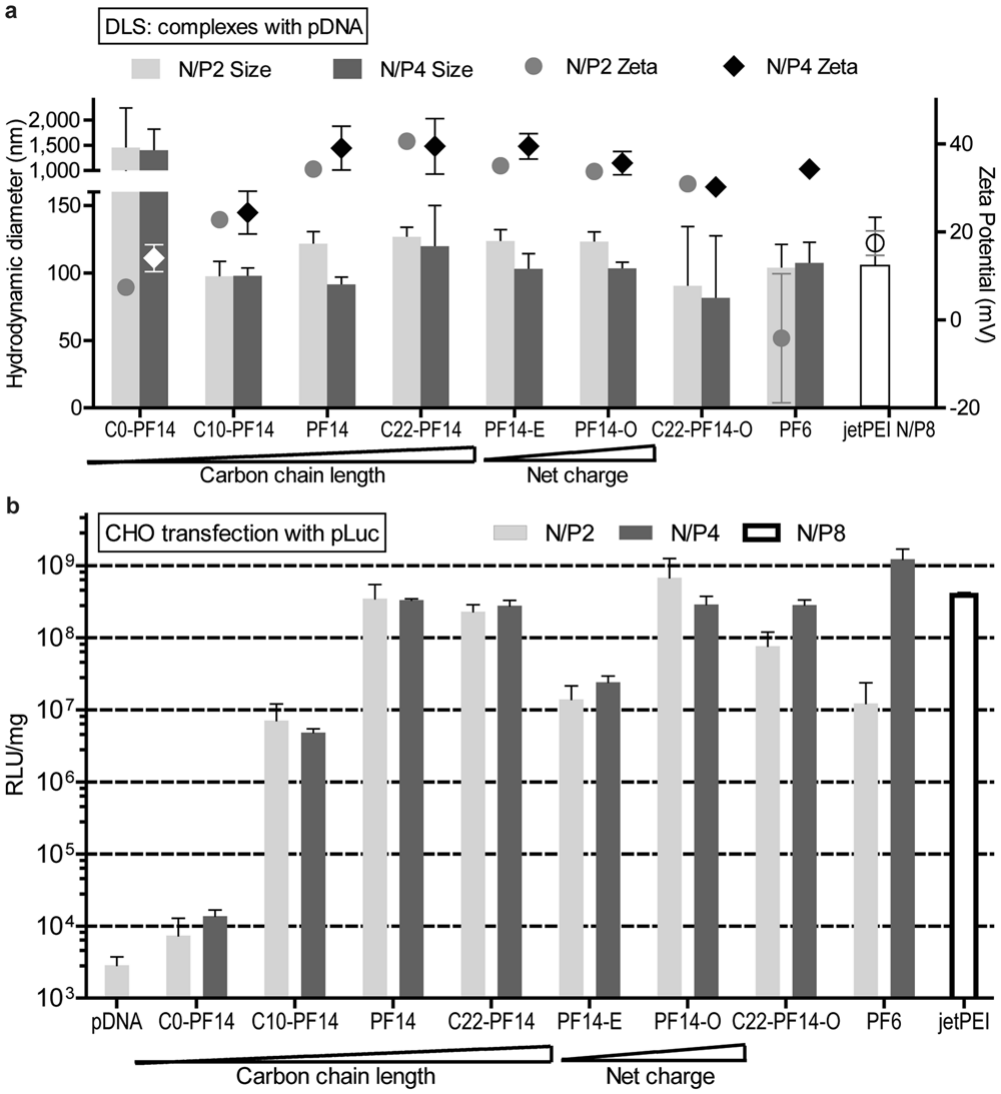
Particle sizes, zeta potentials and transfection efficacies of PF14 analogues with modified charge and hydrophobicity. (**a**) Hydrodynamic diameter and zeta potential of complexes prepared with PF14 analogues and pDNA. (**b**) The pDNA transfection efficacy PF14 and its analogs was evaluated in cultured CHO cells. The most efficient transfection was mediated by the vectors with the highest charge density (PF6 > PF14-O > PF14 > jetPEI). Decrease of the carbon chain length of PF14 also decreased the transfection. Statistical analysis is presented in the Supplementary Data.

We verified that all the charge-modified peptides were able to bind and condense pDNA (Figure S1) and the size and zeta potential were not significantly different from the parent (Fig. 3a, Supplementary Data). The peptides effectively formed similar nanoparticles also in serum environment (Figure S2). When comparing the transfection efficacies, interestingly, the highest gene induction in cell culture was mediated by the vectors that have the highest charge—PF14-O (net charge +7) and PF6 (pH-dependent chargen +10), closely followed by PF14 (+5) and jetPEI (Fig. 3b, Supplementary Data). The lowest efficacy was observed with PF14-E, a CPP with the net charge of +3. It is worth noting that the most efficient transfection formulation—PF14-O at N/P2—utilizes 2.7 times lower concentration of the peptide, compared to the next best formulation (PF14 at N/P4).

### Hydrophobicity of PF14 and surface charge of the particles are directly related

As discussed above, the size and surface charge of the nanoparticles affect their biological effect. We next assessed if modification of the alkyl chain length of PF14 (carbon chain length 18) affects the size and surface charge of the complexes and the resultant efficacy. Therefore, the carbon chain length was increased to 22 (C22-PF14), decreased to 10 (C10-PF14) or eliminated entirely (C0-PF14, Table 1)^18^. All of these analogs were able to bind and condense pDNA effectively into complexes (Figures S1, S2), except for the C10-PF14 (shown in Figure S1) and C0-PF14 (shown in ref.^7^), which were less efficient.

The DLS measured size of the complexes remained generally in the range of 100—130 nm with a positive zeta potential (up to +45 mV) (Fig. 3a). Increasing the N/P slightly decreased the size of the complexes and increased the zeta potential. As an exception, C0-PF14 did not form nanoparticles, instead, micro-sized aggregates with an almost neutral surface charge were formed (Fig. 3a). This observation is supported by our previous work where C0-PF14 complexes were weakly formed and dissociated before uptake by the cells could occur^7^. However, all the analogs with fatty acid modification formed nanoparticles with slightly increased diameter depending on the increased carbon tail length. Interestingly, the zeta potential was also dependent on carbon chain length: the surface charge increased from almost neutral (C0-PF14) to highly positive (PF14 and C22-PF14). The importance of fatty acid modification of the PF14 backbone was also reported in our previous work^18^.

### Hydrophobicity and charge of the complex components affect the stability of nanoparticles and activity towards biological membranes

In order to mediate effective delivery, the nucleic acid content should be protected against degradation, a feature that is likely mediated by the condensation of plasmid molecules into stable nanoparticles. To estimate such stability, we challenged the complexes with different environments that reflect the ability of the peptides to effectively condense and protect the cargo nucleic acid.

We first observed the complexes in serum environment. Incubating the complexes in serum for up to 48 h (Figure S3) showed again that C0-PF14 formed weaker complexes (in agreement with ref.^7^), because the migration of DNA is observed already at 0.5 h in serum, whereas for the other peptides (and jetPEI), less DNA migration can be seen and the onset of migration occurs after 3–6 h incubation in serum. Consequently, C0-PF14 is not able to protect the pDNA from degradation, because the DNA smear can be observed after about 24 h, whereas the naked pDNA is degraded after about 6 h of serum incubation. As to the other peptides, although the accessibility of the DNA to the dye is increased (visible DNA bands in/near the wells), DNA remains within the complex (no migration) (Figure S3). Finally, after 48 h, migration of pDNA from some of the complexes could be observed (PF14, PF14-O, C22-PF14-O) as a result of the serum and/or triton + heparin dissociation, whereas jetPEI, C10-PF14 and C22-PF14 provided less migration and less degradation.

**Figure 4:**
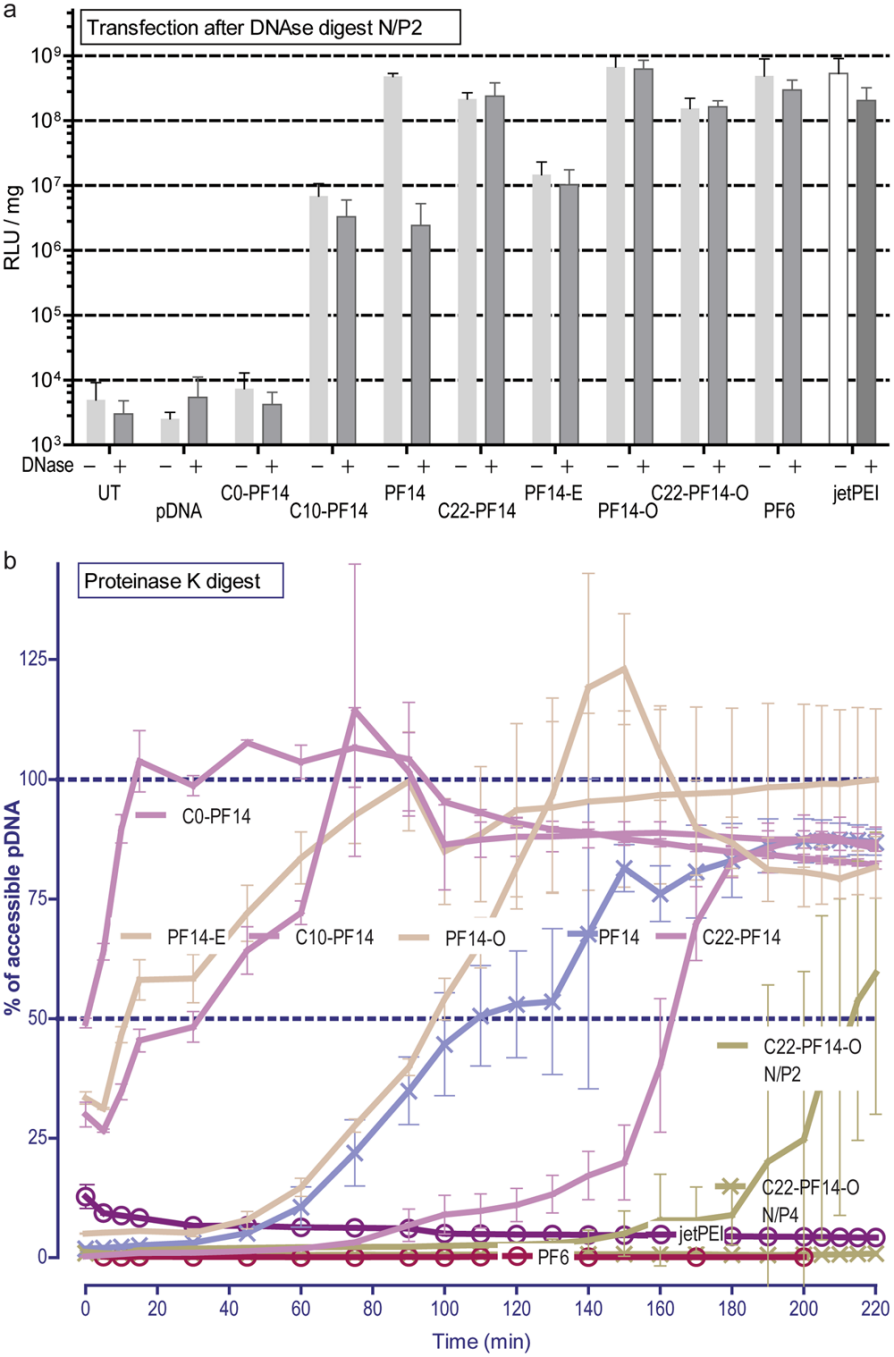
The stability of the complexes in challenging environments. (**a**) Transfection efficacy is assessed after DNase I treatment. The particles are formed at N/P2, digested with DNase and used for the transfection in CHO cells. jetPEI was formed at N/P8. (**b**) The stability of the complexes against proteinase K was evaluated by plotting the amount of accessible pDNA in time (assessed with fluorescent DNA intercalating dye). The peptide complexes were formed at N/P2 (unless indicated otherwise), jetPEI was formed at N/P8.

Next, we attempted to take a more quantitative approach and assess the effect of defined challengers. We subjected the particles to DNase digest. DNase treatment, followed by complex dissociation and DNA quantitation provides direct estimation of the fraction of nucleic acid that is accessible to enzymatic attack. However, we were confronted with a technical challenge with measuring of the amount of undigested intact pDNA, because of the difficulties of dissociating the nanoparticles, especially so because of the N/P ratio increase due to the relative decrease of nucleic acid content (DNA digest) over the peptide content. This was particularly apparent for the complexes with high cationic charge reagents (jetPEI, PF6, C22-PF14-O). To overcome this, we did not attempt to quantify the nucleic acid, but assessed the transfection efficacies, following enzymatic digest of the complexes. Surprisingly, DNase had negligible effect on transfection efficacy at N/P4 (Figure S4) and only minor effects at N/P2 (Fig. 4a). The only peptide apparently susceptible to DNase was, again surprisingly, PF14, which displayed >1 log reduction in transfection efficacy.

We next assessed the vulnerability of the peptide content in the CPP/pDNA complex, using the activity of a proteinase with broad substrate specificity. Digestion of the complexes with proteinase K provided more informative results than with DNase, clearly distinguishing stable particles (formed by PF6) and less stable particles where DNA was liberated into the solution (PF14) (Fig 4b, Table 2). The resistance to proteinase may be mediated both by the condensation effect (i.e. CPP micelle formation and electrostatic binding of nucleic acid) and the susceptibility of the peptide backbone itself (non-natural amino acids and modifications in the peptide may increase resistance). As an example of the latter, PF14 contains ornithines and PF6 has branched lysine with fluoroquinolines^11^, adding considerable bulk of nonpeptide origin. Nevertheless, PF14 backbone can be digested, although it contains non-proteogenic amino acid ornithine, because PF14 analogue without the fatty acid (C0-PF14) is clearly a favorable substrate to the proteinase (Fig. 4b). In addition, incubation of naked peptide solutions (without nucleic acid) with proteinase K show that PF14 is easily degraded (Figure S5), while on the other hand, only negligible effect is observed with PF6. Similar resistance of PF6 (in complex with the pDNA) can be seen in Fig. 4b. Such resistance possibly grants an advantage *in vivo*, likely by increasing the complex stability in blood and resulting in an enhanced transfection.

**Table 2:** Membrane activities (expressed as EC_50_) of CPPs and CPP/pDNA complexes and stabilities against proteinase K treatment (expressed as t_1/2_).

Peptide	EC_50_ (μM) of CPP	EC_50_ (μM) of CPP/pDNA	Proteinase K: t_1/2_ (min) of the complexed pDNA (95%CI)
PF14	0.66	1.43	108.3 (101.9 ± 118.4)
PF14-O	0.60	1.17	94.2 (90.7 ± 97.4)
PF14-E	0.67	2.36	16.7 (10.5 ± 21.8)
C0-PF14	5.36	7.09	0.5 (−0.8 ± 1.5)
C10-PF14	0.36	0.86	26.7 (20.1 ± 32.5)
C22-PF14	0.64	1.47	162.7 (159.6 ± 166.7)
PF6	0.96	1.80	N/A

The proteinase digest also demonstrated that certain modifications in electrostatic and hydrophobic interactions affected the stability of the complex. In one hand, decreasing the CPP charge (PF14-E) rendered the complexes even more susceptible to the proteinase, while increasing the charge (PF14-O) did not improve the stability. Secondly, decreasing the carbon chain length decreased the stability, while using longer fatty acid chain provided higher stability against the digest. Finally, a combination of higher net charge and longer fatty acid (C22-PF14-O), results in significant stability, as the peptide in the particles is now virtually inaccessible to the enzyme, similar to what was observed with PF6 particles.

We also assessed the membrane activities of the CPP nanoparticles and compared these on the basis of EC_50_ (the concentration required to lyse 50% of the cells) (Table 2). The general trend that we observed was that the ranking order of the EC_50_ within the naked CPPs and within the CPP/pDNA complexes was similar: PF6 (or PF6/pDNA) was slightly less active, while PF14 (or PF14/pDNA), along with its charge and hydrophobicity modifications constituted a more active group of peptides, with EC_50_ values about 1.5 times lower, compared to PF6.

Within the PF14 family, the membrane activities correlated with the peptide charge—more cationic CPP had also higher membrane activity. However, when including PF6, a CPP with high positive charge, one can see that the charge alone is probably not the driving factor for the membrane activity. Moreover, the particle surface charges (Fig. 3) and membrane activities do not correlate. The change of PF14 hydrophobicity had stronger impact on EC_50_: PF14 without the carbon chain displayed ~8 times reduction in EC_50_, which probably reflects its poor complex formation ability (Fig. 3 and Figure S1). Surprisingly, PF14 with the carbon chain length 10 (C10-PF14) had the highest membrane activity, making it difficult to relate the membrane activity with any aspects of the nanoparticle formation, particle size or surface charge.

### Systemic gene delivery with new PF14 analogs

**Figure 5:**
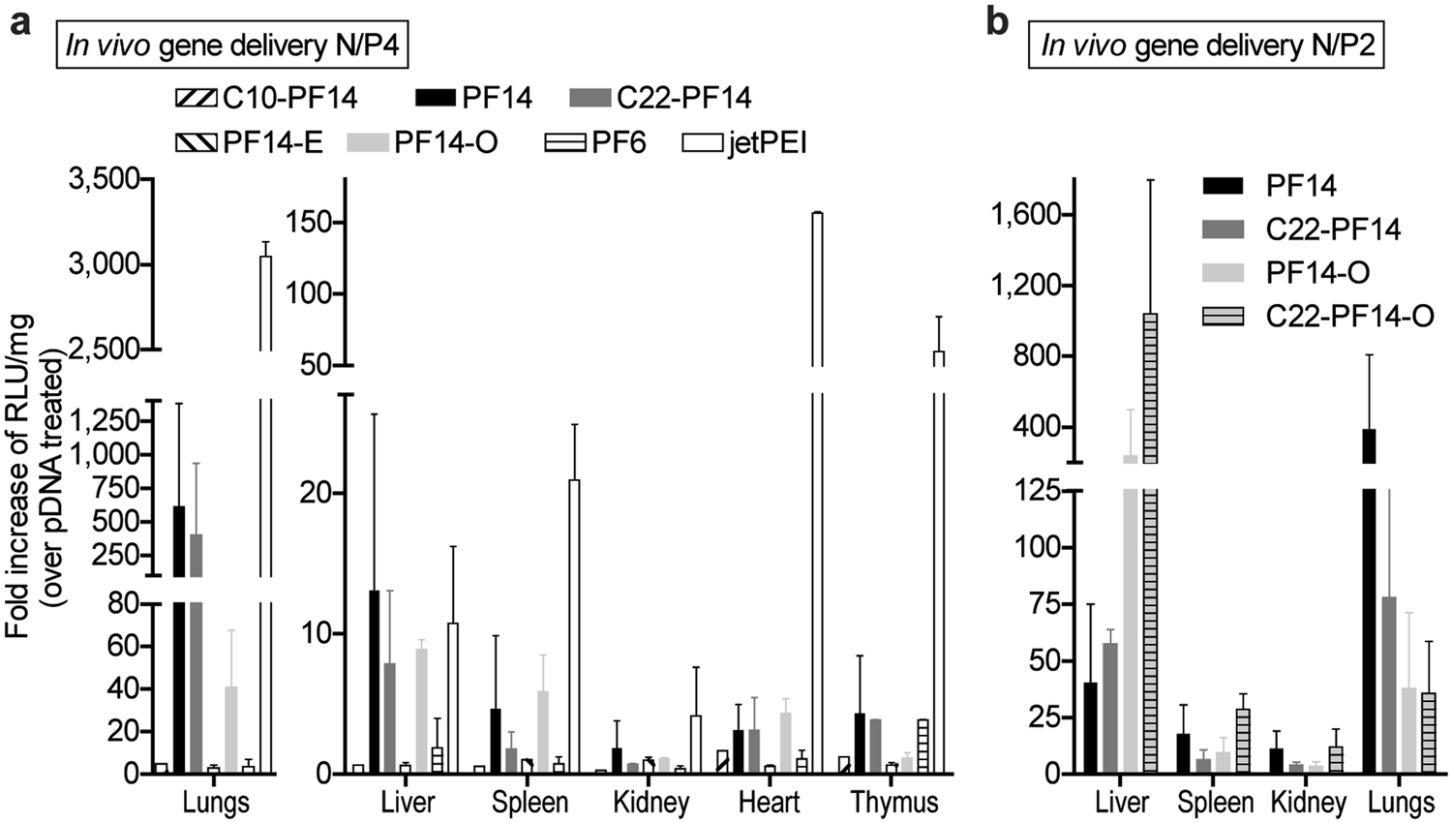
The PF14 analogue C22-PF14-O provides efficient and safe gene delivery *in vivo*. (**a**) Systemic *in vivo* gene delivery efficacies of CPP/pDNA at N/P4, 20 μg pDNA dose. (**b**) The effect of increasing the pDNA dose on *in vivo* gene induction at N/P2 and pDNA 50 μg. Statistical analysis is presented in the Supplementary Data.

After studying the stability and interactions of peptide/pDNA complexes in challenging environments that mimic aspects *in vivo* conditions, we proceeded with the gene induction efficacy assessments after systemic administration in mice. In accordance with previous knowledge with cationic polymers^17^ and PF14^4^, gene delivery effects in the current study could be mainly seen in the lungs and liver, much less in spleen, kidney, and heart (Fig. 5), and insignificant amounts in other tissues (not shown). PF14 and its analogues C22-PF14 and PF14-O were the most efficient mediators of transfection and effectively induced luciferase expression in lungs, liver, and spleen (Fig. 5a). Consistently, the same peptides were also among the top transfection mediators *in vitro* (Fig. 3). Controversially, PF6, which displayed excellent physicochemical properties (Fig. 3a), had a good *in vitro* transfection profile (Fig. 3b), low hemolytic activity (Table 2), and was resistant to enzymatic attack (Fig. 4), was not efficient *in vivo*. Even more curious, PF6 has been shown to be an excellent siRNA delivery vector *in vivo* ^11^, suggesting that different delivery bottlenecks exist for the plasmid versus siRNA delivery. To gauge the subpar performance of PF6 further, we increased the levels of the peptide and administered PF6/pDNA up to very high ratio (N/P13). Remarkably, no side effects occurred even at these high ratios (zero lethality), but gene delivery efficacy did not improve (data not shown). It may be that PF6 forms extremely stable and strong particles that are resistant to enzymatic attack yet are also not able to dissociate and release the nucleic acid, at least in the form of plasmid DNA.

A commercial reagent jetPEI, which was among the best performers in cell culture transfection, was the most efficient carrier also *in vivo* (Fig. 5a, Supplementary Data). Because the components of the reagent are not known, we tried a more direct comparison between the CPPs and a linear PEI (22 kDa) polymer. However, acute toxicity was observed (immediate lethal effects in two animals from two injections). This suggests that further formulation optimization is warranted for PEI and possibly also for our CPPs.

Finally, we assessed the *in vivo* performance of the PF14 charge and fatty acid modified peptides in the delivery of higher amounts of pDNA. Inspired by the formulation of PF14 at lower N/P, which resulted in increased safety and efficient transfection (Fig. 2), we applied the same strategy for the PF14 analogs with increased hydrophobicity and charge. Interestingly, both modifications, C22-PF14 and PF14-O were more efficient in liver transfection, whereas less efficiency was seen in lung (Fig. 5b, Supplementary Data). We also assessed the performance of both modifications at the same time and saw that C22-PF14-O liver transfection efficacy was augmented even further. Again, as was the case with PF14 at lower N/P ratios, no toxic effects were observed (lethality in 0 cases).

## Conclusions

Safety and efficacy are major factors to be considered in nucleic acid delivery in gene therapeutic applications. In the current study we have assessed important parameters in pDNA vectorization that shed light to these factors. We have optimized the nanoparticle formulation of our efficient CPP, PF14, for the delivery of pDNA, resulting in effective and nontoxic gene delivery. Accordingly, efficient lung transfection with PF14 was achieved, which can be potentially used for the lung-targeted gene therapy. Secondly, based on current studies on nanoparticle formation, we have introduced modifications in the CPP and developed more efficient *in vivo* gene delivery vector C22-PF14-O, where its advantage in liver transfection is of notable interest. Combined with the optimized formulation, both PF14 and the novel C22-PF14-O allow efficient transfection of lung and liver without toxic side effects, which will be important properties when choosing a delivery vector for gene therapeutic targeting of diseases associated with these organs.

## Electronic supplementary material

Supplementary material
