# Supplementary material for: Effective in vivo gene delivery with reduced toxicity, achieved by charge and fatty acid -modified cell penetrating peptide
Source: Sci Rep. 2017 Dec 6;7:17056. doi: 10.1038/s41598-017-17316-y (PMC5719086; doi:10.1038/s41598-017-17316-y)
Supplement: Supplementary file 1 — Supplementary material [file 41598_2017_17316_MOESM1_ESM.pdf]

Effective *in vivo* gene delivery with reduced toxicity, achieved by charge and fatty acid -modified cell penetrating peptide

Kaido Kurrikoff, Kadi-Liis Veiman, Kadri Künnapuu, Elin Madli Peets, Tõnis Lehto, Ly Pärnaste, Piret Arukuusk, Ülo Langel

Supplementary Figures and Supplementary Data

Supplementary Figures

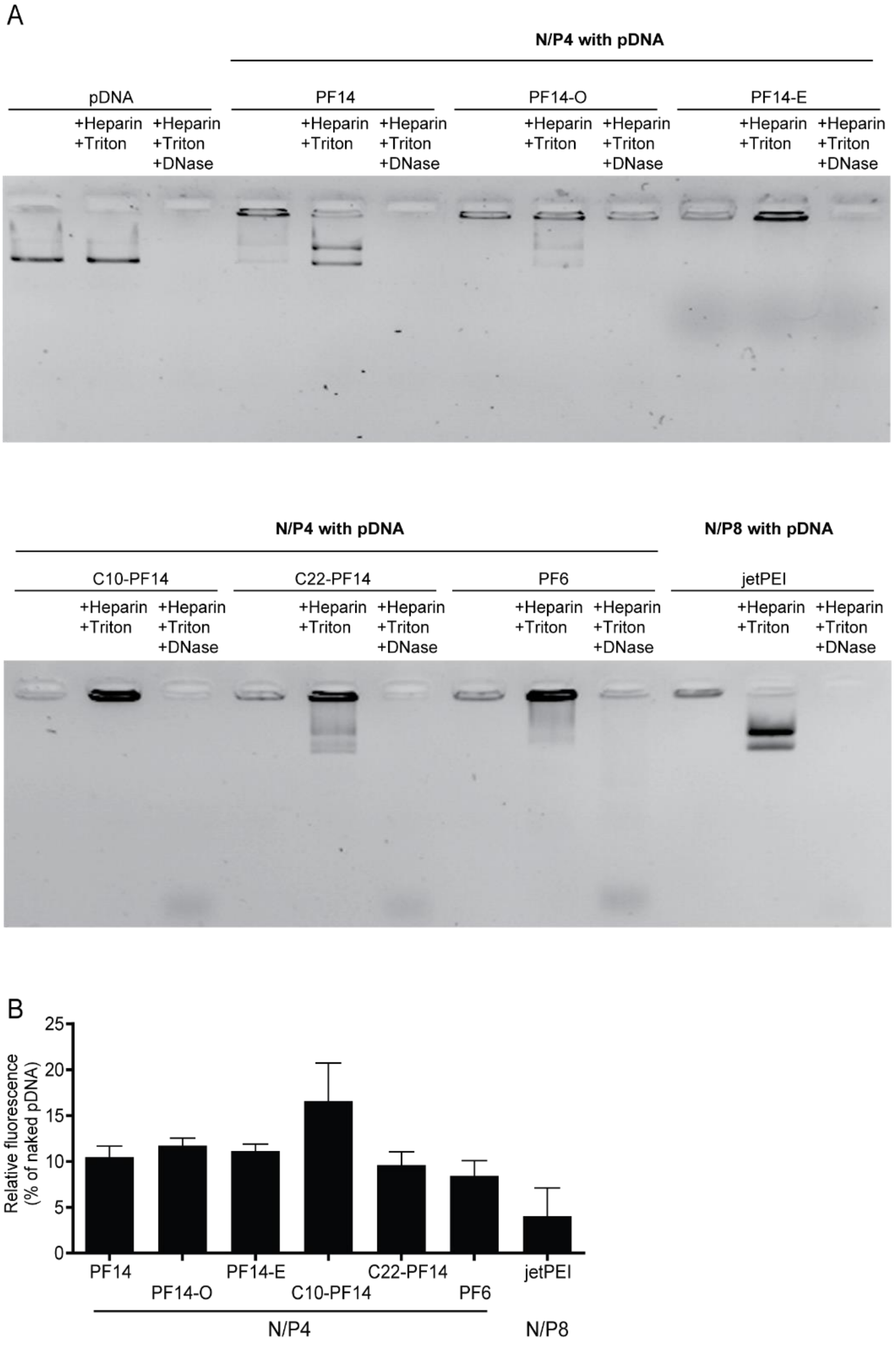

Figure S1. The complex formation between PF14 analogues and pDNA, assessed by gel shift and EtBr exclusion assay. The pDNA binding was assessed by (**a**) gel shift assay and (**b**) by EtBr exclusion assay.

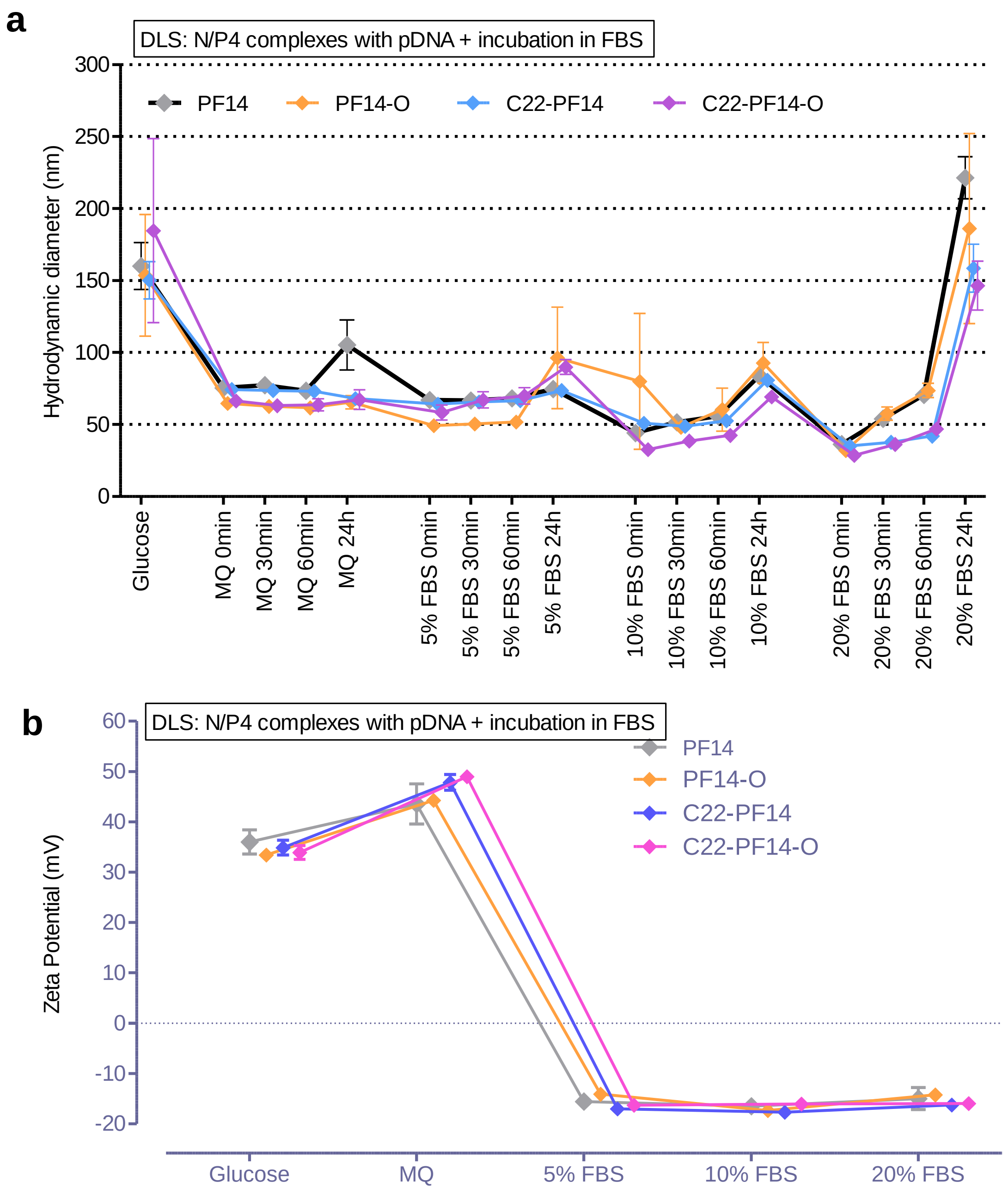

Figure S2. DLS of PF14 analogues with modified charge and hydrophobicity, complexed with pDNA at N/P4, after incubation in FBS. The complexes of the peptides and pDNA (N/P4) were formulated in water (MQ), 5% glucose, and FBS. (**a**) Average hydrodynamic diameter after incubation in different environments for 0, 30, 60 min and 24h. (**b**) Zeta potential in MQ, 5% glucose and FBS.

0.5 h incubation in FBS

Ladder	C0-PF14				C10-PF14				PF14				C22-PF14				pDNA	MQ	pDNA
	-FBS		+FBS		-FBS		+FBS		-FBS		+FBS		-FBS		+FBS		+FBS	+FBS	-FBS
	+Triton +heparin	-	+Triton +heparin	-	+Triton +heparin	-	+Triton +heparin	-	+Triton +heparin	-	+Triton +heparin	-	+Triton +heparin	-	+Triton +heparin	-	-	-	-

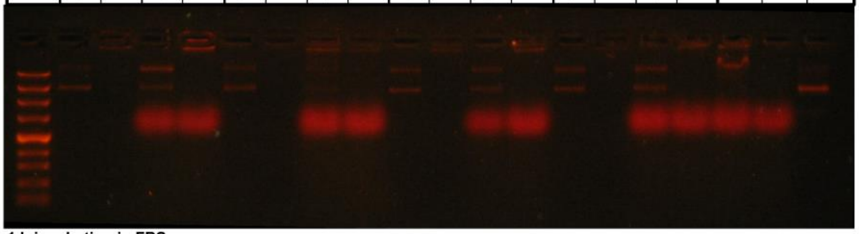

1 h incubation in FBS

Ladder	C0-PF14				C10-PF14				PF14				C22-PF14				pDNA		MQ
	-FBS		+FBS		-FBS		+FBS		-FBS		+FBS		-FBS		+FBS		+FBS	-FBS	+FBS
	+Triton +heparin	-	+Triton +heparin	-	+Triton +heparin	-	+Triton +heparin	-	+Triton +heparin	-	+Triton +heparin	-	+Triton +heparin	-	+Triton +heparin	-	-	-	-

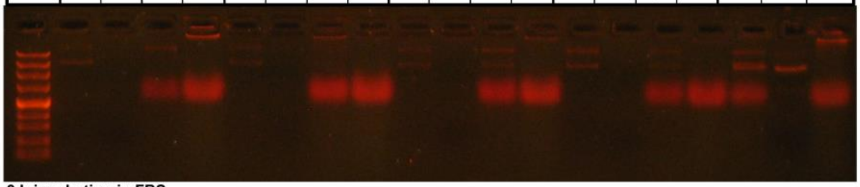

3 h incubation in FBS

Ladder	C0-PF14				C10-PF14				PF14				C22-PF14				pDNA		MQ
	-FBS		+FBS		-FBS		+FBS		-FBS		+FBS		-FBS		+FBS		-FBS	+FBS	+FBS
	+Triton +heparin	-	+Triton +heparin	-	+Triton +heparin	-	+Triton +heparin	-	+Triton +heparin	-	+Triton +heparin	-	+Triton +heparin	-	+Triton +heparin	-	-	-	-

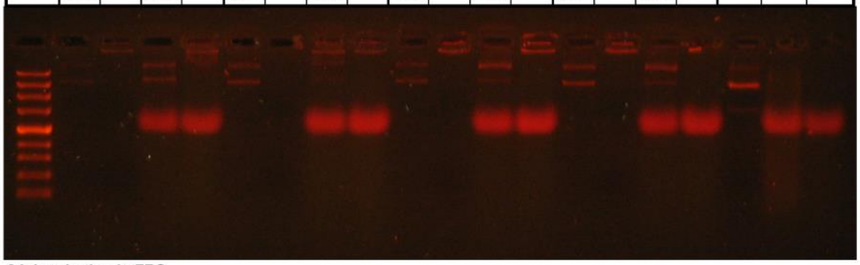

6 h incubation in FBS

Ladder	C0-PF14				C10-PF14				PF14				C22-PF14				pDNA		MQ
	-FBS		+FBS		-FBS		+FBS		-FBS		+FBS		-FBS		+FBS		-FBS	+FBS	+FBS
	+Triton +heparin	-	+Triton +heparin	-	+Triton +heparin	-	+Triton +heparin	-	+Triton +heparin	-	+Triton +heparin	-	+Triton +heparin	-	+Triton +heparin	-	-	-	-

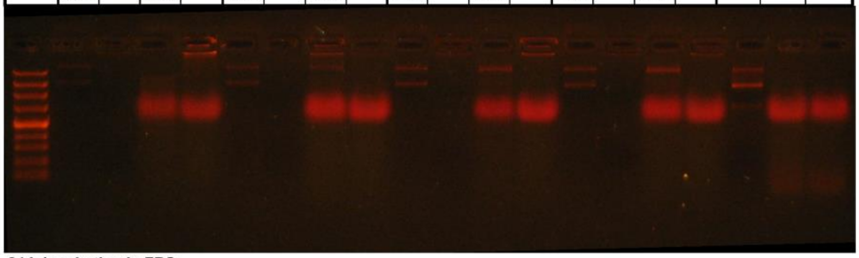

24 h incubation in FBS

Ladder	C0-PF14				C10-PF14				PF14				C22-PF14				pDNA		MQ
	-FBS		+FBS		-FBS		+FBS		-FBS		+FBS		-FBS		+FBS		-FBS	-FBS	+FBS
	+Triton +heparin	-	+Triton +heparin	-	+Triton +heparin	-	+Triton +heparin	-	+Triton +heparin	-	+Triton +heparin	-	+Triton +heparin	-	+Triton +heparin	-	-	+Triton +heparin	-

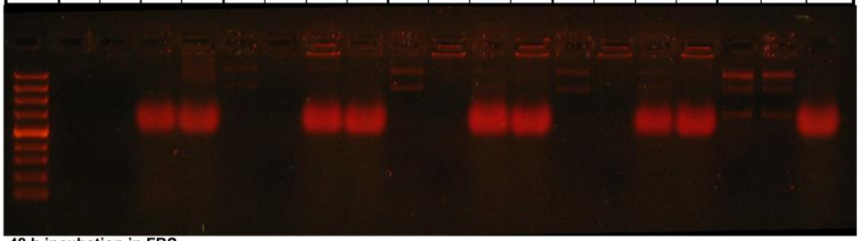

48 h incubation in FBS

Ladder	C0-PF14				C10-PF14				PF14				C22-PF14				pDNA		MQ
	-FBS		+FBS		-FBS		+FBS		-FBS		+FBS		-FBS		+FBS		-FBS	-FBS	+FBS
	+Triton +heparin	-	+Triton +heparin	-	+Triton +heparin	-	+Triton +heparin	-	+Triton +heparin	-	+Triton +heparin	-	+Triton +heparin	-	+Triton +heparin	-	-	-	-

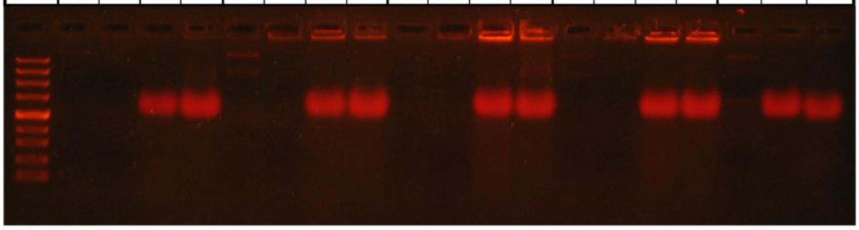

0.5 h incubation in FBS

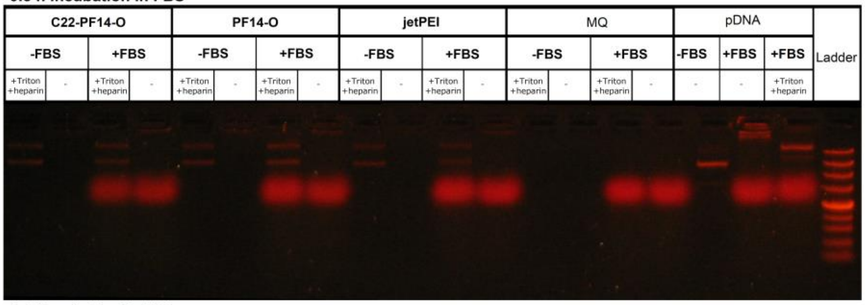

1 h incubation in FBS

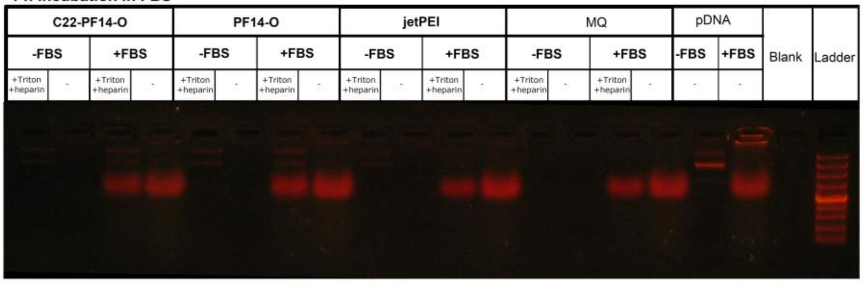

3 h incubation in FBS

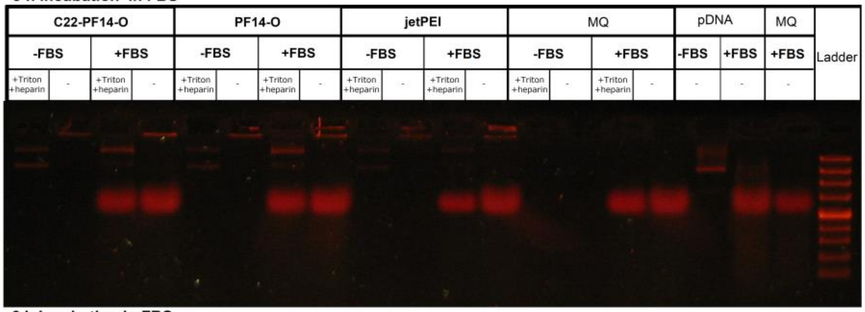

6 h incubation in FBS

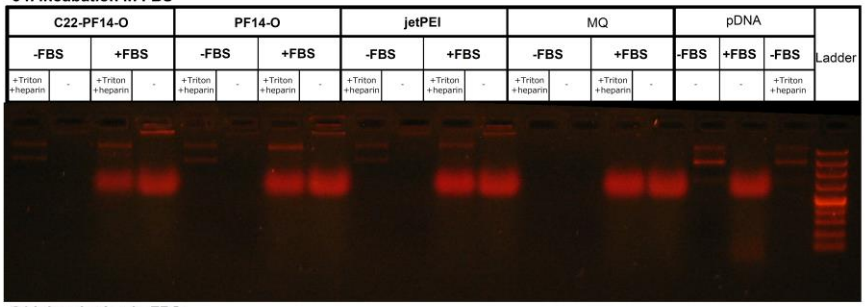

24 h incubation in FBS

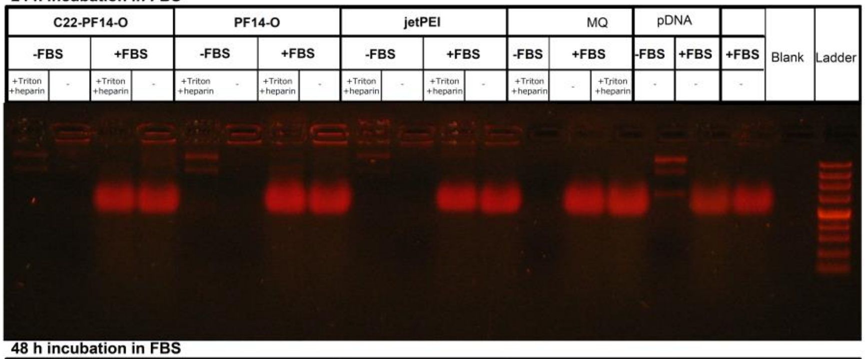

48 h incubation in FBS

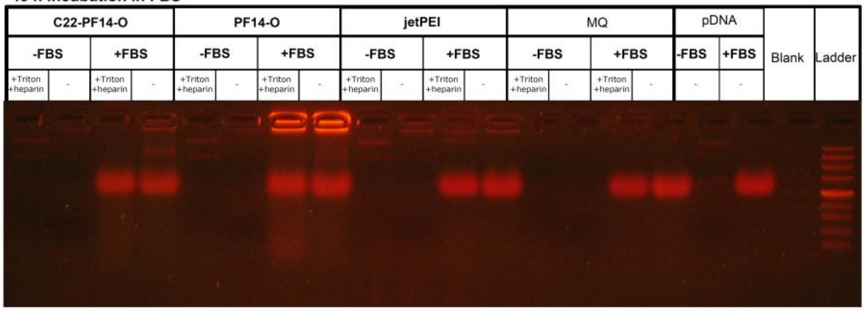

Figure S3. The stability of the complexes after incubation in FBS, visualized by gel shift assay. The incubation was carried out for 0.5, 1, 3, 6, 24 and 48h in 50% FBS, followed by dissociation with Triton X-100 and heparin and gel electrophoresis.

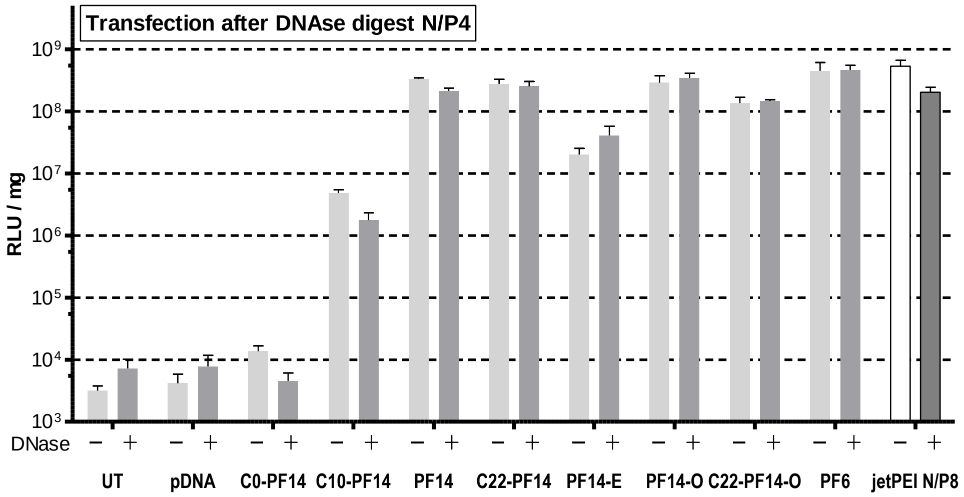

Figure S4. Transfection efficacy, assessed after challenge with DNAse I. The PF14/pLuc complexes were formed at N/P4, digested with DNAse and used for the transfection in CHO cells.

A

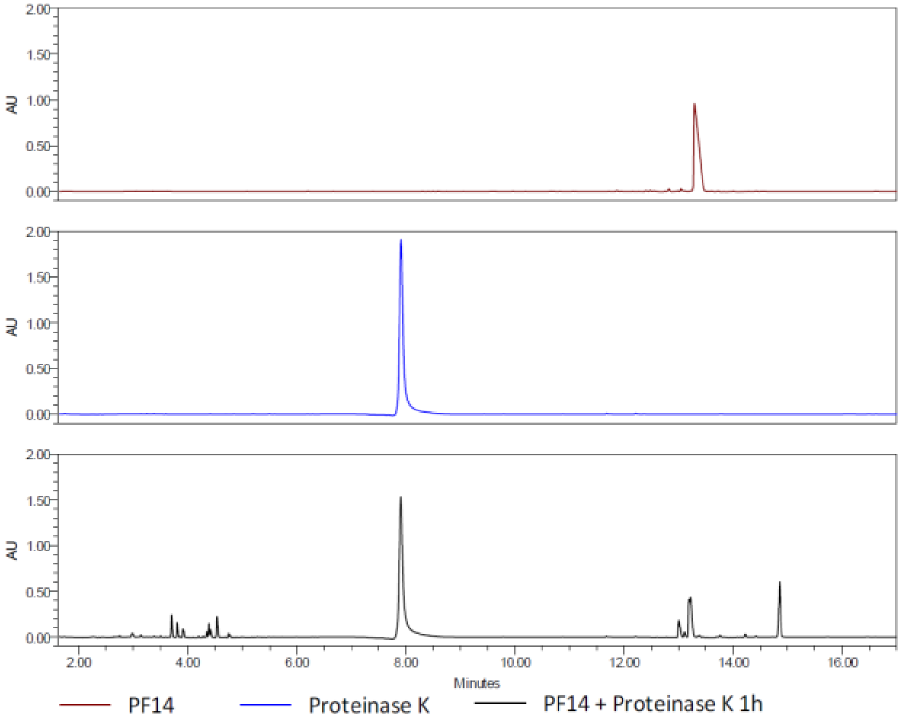

B

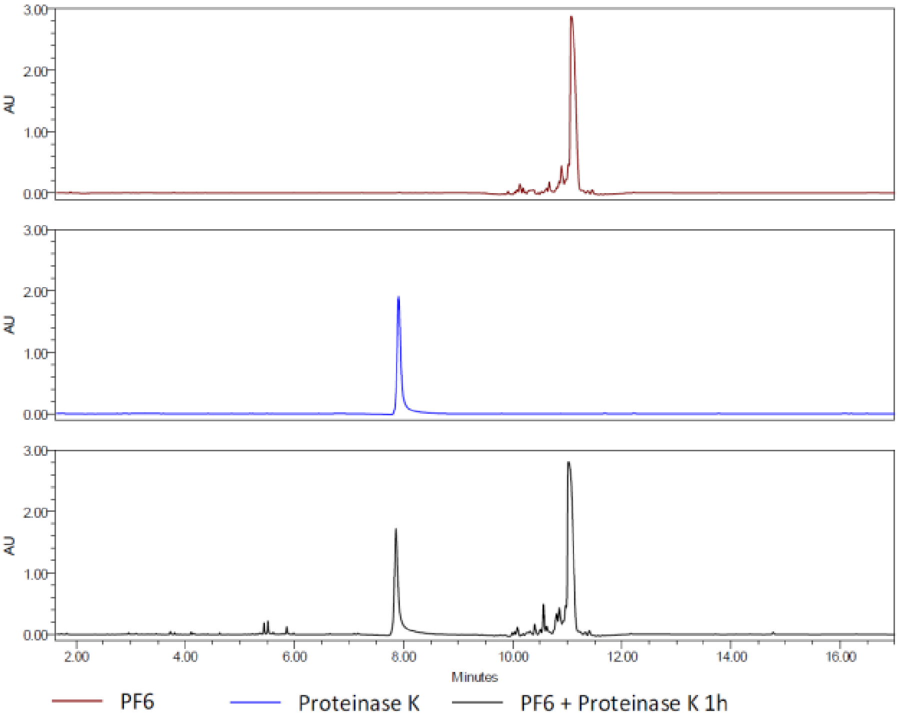

Figure S5. CPP digestion by proteinase K treatment. 1x proteinase K was incubated with 1.5x excess volume of (**a**) 100 µM PF14 and (**b**) 100 µM of PF6 at 37°C for 1 hour. After incubation, the peptide-proteinase K mixture was analyzed by UPLC (Waters), using C18 column and 5-95% acetonitrile (0.1% TFA) gradient.

Supplementary Data

Statistical analysis was performed using Statistica software (Dell Inc. (2015). Dell Statistica (data analysis software system), version 13. software.dell.com).

Statistical analysis of data presented in Figure 3a

ANOVA ("peptide" x "N/P ratio"), followed by Tukey post hoc. Note: because jetPEI was formulated only at single N/P, jetPEI was not included into the two-way ANOVA.

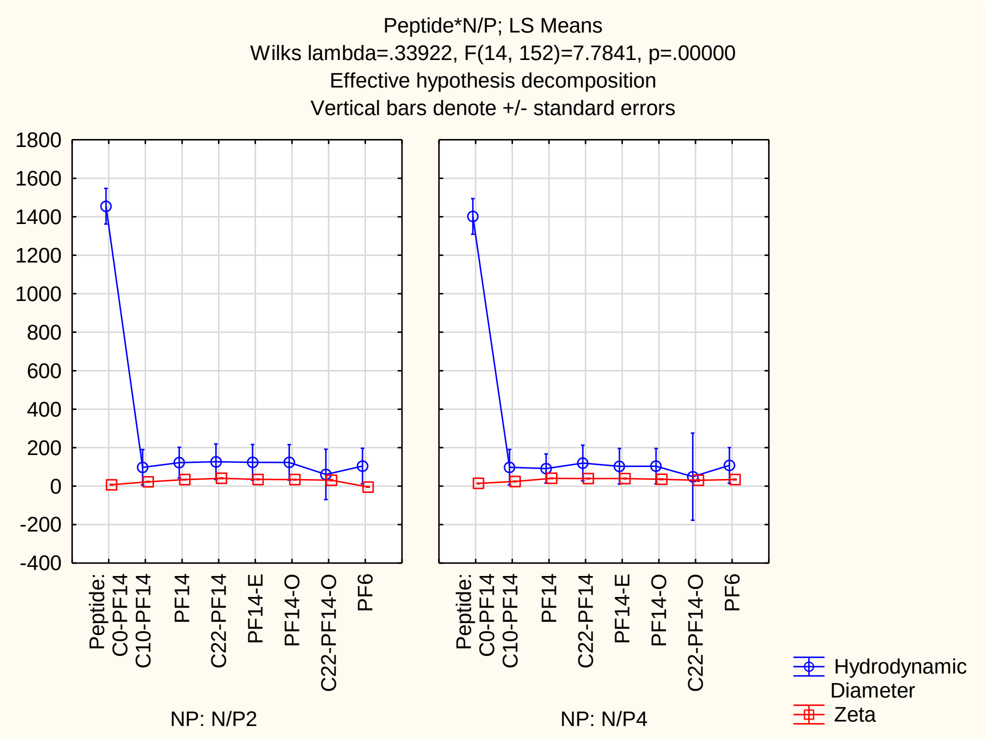

Effect	Multivariate Tests of Significance (Fig3A)<br>Sigma-restricted parameterization<br>Effective hypothesis decomposition<br>Test	Value	F	Effect df	Error df	p
Intercept	Wilks	0.028293	1305.102	2	76	0.000000
Peptide	Wilks	0.035451	46.807	14	152	0.000000
NP	Wilks	0.646476	20.780	2	76	0.000000
Peptide*NP	Wilks	0.339222	7.784	14	152	0.000000

Tukey HSD test; variable Hydrodynamic Diameter (Fig3A)
Approximate Probabilities for Post Hoc Tests
Error: Between MS = 51384., df = 77.000

Cell No.	Peptide	NP	{1} 1454.7	{2} 1401.8	{3} 97.450	{4} 97.967	{5} 121.71	{6} 91.509	{7} 126.80	{8} 119.77	{9} 123.55	{10} 102.92	{11} 123.20	{12} 103.32	{13} 60.833	{14} 49.050	{15} 103.93	{16} 107.47
1	C0-PF14	N/P2		1.00	0.00	0.00	0.00	0.00	0.00	0.00	0.00	0.00	0.00	0.00	0.00	0.00	0.00	0.00
2	C0-PF14	N/P4	1.00		0.00	0.00	0.00	0.00	0.00	0.00	0.00	0.00	0.00	0.00	0.00	0.00	0.00	0.00
3	10-PF14	N/P2	0.00	0.00		1.00	1.00	1.00	1.00	1.00	1.00	1.00	1.00	1.00	1.00	1.00	1.00	1.00
4	10-PF14	N/P4	0.00	0.00	1.00		1.00	1.00	1.00	1.00	1.00	1.00	1.00	1.00	1.00	1.00	1.00	1.00
5	PF14	N/P2	0.00	0.00	1.00	1.00		1.00	1.00	1.00	1.00	1.00	1.00	1.00	1.00	1.00	1.00	1.00
6	PF14	N/P4	0.00	0.00	1.00	1.00	1.00		1.00	1.00	1.00	1.00	1.00	1.00	1.00	1.00	1.00	1.00
7	22-PF14	N/P2	0.00	0.00	1.00	1.00	1.00	1.00		1.00	1.00	1.00	1.00	1.00	1.00	1.00	1.00	1.00
8	22-PF14	N/P4	0.00	0.00	1.00	1.00	1.00	1.00	1.00		1.00	1.00	1.00	1.00	1.00	1.00	1.00	1.00
9	PF14-E	N/P2	0.00	0.00	1.00	1.00	1.00	1.00	1.00	1.00		1.00	1.00	1.00	1.00	1.00	1.00	1.00
10	PF14-E	N/P4	0.00	0.00	1.00	1.00	1.00	1.00	1.00	1.00	1.00		1.00	1.00	1.00	1.00	1.00	1.00
11	PF14-O	N/P2	0.00	0.00	1.00	1.00	1.00	1.00	1.00	1.00	1.00	1.00		1.00	1.00	1.00	1.00	1.00
12	PF14-O	N/P4	0.00	0.00	1.00	1.00	1.00	1.00	1.00	1.00	1.00	1.00	1.00		1.00	1.00	1.00	1.00
13	2-PF14-O	N/P2	0.00	0.00	1.00	1.00	1.00	1.00	1.00	1.00	1.00	1.00	1.00	1.00		1.00	1.00	1.00
14	2-PF14-O	N/P4	0.00	0.00	1.00	1.00	1.00	1.00	1.00	1.00	1.00	1.00	1.00	1.00	1.00		1.00	1.00
15	PF6	N/P2	0.00	0.00	1.00	1.00	1.00	1.00	1.00	1.00	1.00	1.00	1.00	1.00	1.00	1.00		1.00
16	PF6	N/P4	0.00	0.00	1.00	1.00	1.00	1.00	1.00	1.00	1.00	1.00	1.00	1.00	1.00	1.00	1.00	

Tukey HSD test; variable Zeta (Fig3A)
Approximate Probabilities for Post Hoc Tests
Error: Between MS = 22.609, df = 77.000

Cell No.	Peptide	NP	{1} 7.4367	{2} 14.050	{3} 22.733	{4} 24.367	{5} 33.813	{6} 40.656	{7} 40.583	{8} 39.417	{9} 35.017	{10} 39.417	{11} 33.717	{12} 35.600	{13} 30.933	{14} 30.200	{15} -4.168	{16} 34.267
1	C0-PF14	N/P2		0.55	0.00	0.00	0.00	0.00	0.00	0.00	0.00	0.00	0.00	0.00	0.00	0.00	0.01	0.00
2	C0-PF14	N/P4	0.55		0.13	0.03	0.00	0.00	0.00	0.00	0.00	0.00	0.00	0.00	0.00	0.14	0.00	0.00
3	C10-PF14	N/P2	0.00	0.13		1.00	0.00	0.00	0.00	0.00	0.00	0.00	0.01	0.00	0.53	0.98	0.00	0.01
4	C10-PF14	N/P4	0.00	0.03	1.00		0.03	0.00	0.00	0.00	0.02	0.00	0.07	0.01	0.84	1.00	0.00	0.04
5	PF14	N/P2	0.00	0.00	0.00	0.03		0.21	0.39	0.71	1.00	0.71	1.00	1.00	1.00	1.00	0.00	1.00
6	PF14	N/P4	0.00	0.00	0.00	0.00	0.21		1.00	1.00	0.66	1.00	0.31	0.81	0.17	0.77	0.00	0.45
7	C22-PF14	N/P2	0.00	0.00	0.00	0.00	0.39	1.00		1.00	0.80	1.00	0.48	0.90	0.25	0.81	0.00	0.62
8	C22-PF14	N/P4	0.00	0.00	0.00	0.00	0.71	1.00	1.00		0.96	1.00	0.77	0.99	0.47	0.91	0.00	0.88
9	PF14-E	N/P2	0.00	0.00	0.00	0.02	1.00	0.66	0.80	0.96		0.96	1.00	1.00	1.00	1.00	0.00	1.00
10	PF14-E	N/P4	0.00	0.00	0.00	0.00	0.71	1.00	1.00	1.00	0.96		0.77	0.99	0.47	0.91	0.00	0.88
11	PF14-O	N/P2	0.00	0.00	0.01	0.07	1.00	0.31	0.48	0.77	1.00	0.77		1.00	1.00	1.00	0.00	1.00
12	PF14-O	N/P4	0.00	0.00	0.00	0.01	1.00	0.81	0.90	0.99	1.00	0.99	1.00		0.99	1.00	0.00	1.00
13	C22-PF14-O	N/P2	0.00	0.00	0.53	0.84	1.00	0.17	0.25	0.47	1.00	0.47	1.00	0.99		1.00	0.00	1.00
14	C22-PF14-O	N/P4	0.00	0.14	0.98	1.00	1.00	0.77	0.81	0.91	1.00	0.91	1.00	1.00	1.00		0.00	1.00
15	PF6	N/P2	0.01	0.00	0.00	0.00	0.00	0.00	0.00	0.00	0.00	0.00	0.00	0.00	0.00	0.00		0.00
16	PF6	N/P4	0.00	0.00	0.01	0.04	1.00	0.45	0.62	0.88	1.00	0.88	1.00	1.00	1.00	1.00	0.00	

Tukey HSD test; variable Hydrodynamic Diameter (Fig3A)
Approximate Probabilities for Post Hoc Tests
Error: Between MS = 51384., df = 77.000

Cell No.	Peptide	{1} 1428.3	{2} 97.708	{3} 105.72	{4} 123.28	{5} 113.23	{6} 113.26	{7} 57.887	{8} 105.70
1	C0-PF14		0.00	0.00	0.00	0.00	0.00	0.00	0.00
2	C10-PF14	0.00		1.00	1.00	1.00	1.00	1.00	1.00
3	PF14	0.00	1.00		1.00	1.00	1.00	1.00	1.00
4	C22-PF14	0.00	1.00	1.00		1.00	1.00	1.00	1.00
5	PF14-E	0.00	1.00	1.00	1.00		1.00	1.00	1.00
6	PF14-O	0.00	1.00	1.00	1.00	1.00		1.00	1.00
7	C22-PF14-O	0.00	1.00	1.00	1.00	1.00	1.00		1.00
8	PF6	0.00	1.00	1.00	1.00	1.00	1.00	1.00	

Tukey HSD test; variable Zeta (Fig3A)
Approximate Probabilities for Post Hoc Tests
Error: Between MS = 22.609, df = 77.000

Cell No.	Peptide	{1} 10.743	{2} 23.550	{3} 37.435	{4} 40.000	{5} 37.217	{6} 34.658	{7} 30.750	{8} 15.049
1	C0-PF14		0.00	0.00	0.00	0.00	0.00	0.00	0.35
2	C10-PF14	0.00		0.00	0.00	0.00	0.00	0.16	0.00
3	PF14	0.00	0.00		0.84	1.00	0.78	0.20	0.00
4	C22-PF14	0.00	0.00	0.84		0.84	0.12	0.02	0.00
5	PF14-E	0.00	0.00	1.00	0.84		0.89	0.28	0.00
6	PF14-O	0.00	0.00	0.78	0.12	0.89		0.84	0.00
7	C22-PF14-O	0.00	0.16	0.20	0.02	0.28	0.84		0.00
8	PF6	0.35	0.00	0.00	0.00	0.00	0.00	0.00	

Statistical analysis of data presented in Figure 3b

1-way ANOVA, followed by Tukey post hoc.

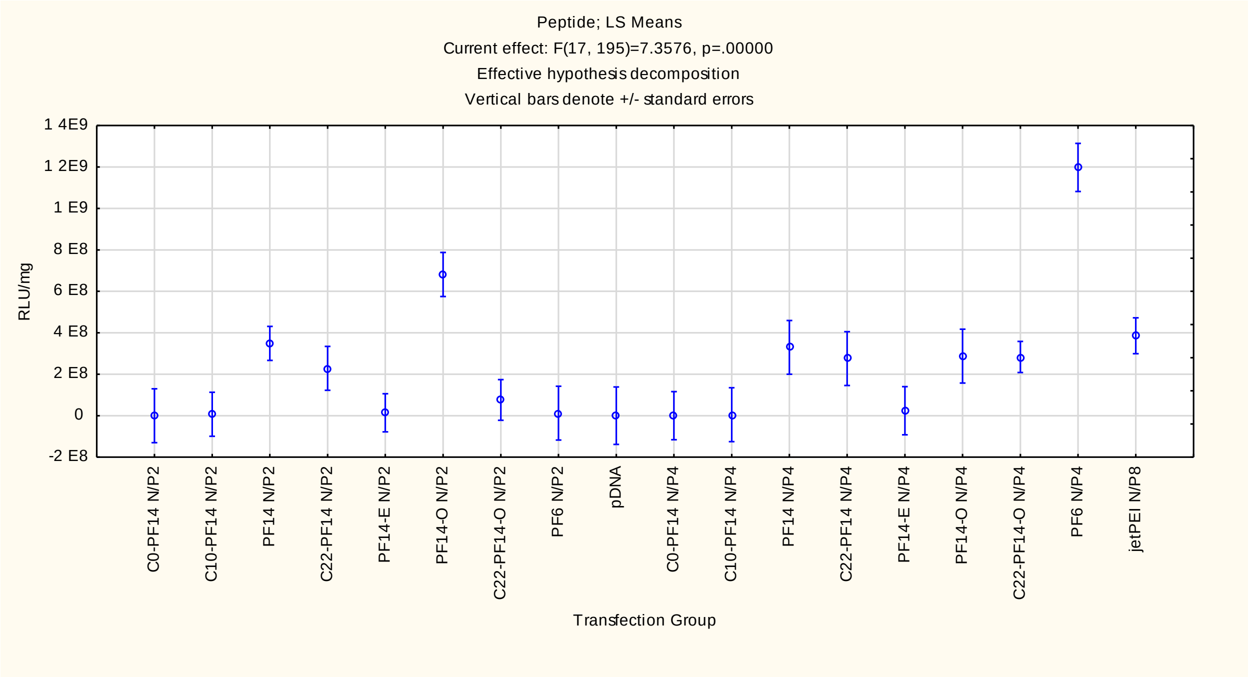

	Univariate Tests of Significance for RLU/mg (Fig3B statistics.sta)<br>Sigma-restricted parameterization<br>Effective hypothesis decomposition				
Effect	SS	Degr. of Freedom	MS	F	p
Intercept	1.002213E+19	1	1.002213E+19	74 .27940	0 .000000
Peptide	1.687621E+19	17	9.927180E+17	7 .35757	0 .000000
Error	2.631033E+19	195	1.349248E+17		

Tukey HSD test; variable RLU/mg (Fig3B statistics.sta)
Approximate Probabilities for Post Hoc Tests
Error: Between MS = 135E15, df = 195.00

Cell No.	Transfection group	{1} 7357.5	{2} 7052E3	{3} 3488E5	{4} 2286E5	{5} 1390E4	{6} 6814E5	{7} 7584E4	{8} 1227E4	{9} 2855.9	{10} 13652.	{11} 4811E3	{12} 3297E5	{13} 2757E5	{14} 2400E4	{15} 2876E5	{16} 2831E5	{17} 1198E6	{18} 3859E5
1	C0-PF14 N/P2		1 .00	0 .71	1 .00	1 .00	0 .01	1 .00	1 .00	1 .00	1 .00	1 .00	0 .95	0 .99	1 .00	0 .99	0 .92	0 .00	0 .55
2	C10-PF14 N/P2	1 .00		0 .49	0 .99	1 .00	0 .00	1 .00	1 .00	1 .00	1 .00	1 .00	0 .90	0 .98	1 .00	0 .97	0 .80	0 .00	0 .33
3	PF14 N/P2	0 .71	0 .49		1 .00	0 .37	0 .55	0 .80	0 .76	0 .78	0 .57	0 .73	1 .00	1 .00	0 .70	1 .00	1 .00	0 .00	1 .00
4	C22-PF14 N/P2	1 .00	0 .99	1 .00		0 .99	0 .19	1 .00	1 .00	1 .00	0 .99	1 .00	1 .00	1 .00	1 .00	1 .00	1 .00	0 .00	1 .00
5	PF14-E N/P2	1 .00	1 .00	0 .37	0 .99		0 .00	1 .00	1 .00	1 .00	1 .00	1 .00	0 .88	0 .98	1 .00	0 .96	0 .70	0 .00	0 .23
6	PF14-O N/P2	0 .01	0 .00	0 .55	0 .19	0 .00		0 .00	0 .01	0 .01	0 .00	0 .01	0 .82	0 .59	0 .00	0 .65	0 .17	0 .09	0 .78
7	C22-PF14-O N/P2	1 .00	1 .00	0 .80	1 .00	1 .00	0 .00		1 .00	1 .00	1 .00	1 .00	0 .99	1 .00	1 .00	1 .00	0 .97	0 .00	0 .63
8	PF6 N/P2	1 .00	1 .00	0 .76	1 .00	1 .00	0 .01	1 .00		1 .00	1 .00	1 .00	0 .96	0 .99	1 .00	0 .99	0 .94	0 .00	0 .61
9	pDNA	1 .00	1 .00	0 .78	1 .00	1 .00	0 .01	1 .00	1 .00		1 .00	1 .00	0 .96	0 .99	1 .00	0 .99	0 .95	0 .00	0 .64
10	C0-PF14 N/P4	1 .00	1 .00	0 .57	0 .99	1 .00	0 .00	1 .00	1 .00	1 .00		1 .00	0 .92	0 .98	1 .00	0 .98	0 .85	0 .00	0 .41
11	C10-PF14 N/P4	1 .00	1 .00	0 .73	1 .00	1 .00	0 .01	1 .00	1 .00	1 .00	1 .00		0 .95	0 .99	1 .00	0 .99	0 .93	0 .00	0 .58
12	PF14 N/P4	0 .95	0 .90	1 .00	1 .00	0 .88	0 .82	0 .99	0 .96	0 .96	0 .92	0 .95		1 .00	0 .96	1 .00	1 .00	0 .00	1 .00
13	C22-PF14 N/P4	0 .99	0 .98	1 .00	1 .00	0 .98	0 .59	1 .00	0 .99	0 .99	0 .98	0 .99	1 .00		0 .99	1 .00	1 .00	0 .00	1 .00
14	PF14-E N/P4	1 .00	1 .00	0 .70	1 .00	1 .00	0 .00	1 .00	1 .00	1 .00	1 .00	1 .00	0 .96	0 .99		0 .99	0 .92	0 .00	0 .53
15	PF14-O N/P4	0 .99	0 .97	1 .00	1 .00	0 .96	0 .65	1 .00	0 .99	0 .99	0 .98	0 .99	1 .00	1 .00	0 .99		1 .00	0 .00	1 .00
16	C22-PF14-O N/P4	0 .92	0 .80	1 .00	1 .00	0 .70	0 .17	0 .97	0 .94	0 .95	0 .85	0 .93	1 .00	1 .00	0 .92	1 .00		0 .00	1 .00
17	PF6 N/P4	0 .00	0 .00	0 .00	0 .00	0 .00	0 .09	0 .00	0 .00	0 .00	0 .00	0 .00	0 .00	0 .00	0 .00	0 .00	0 .00		0 .00
18	jetPEI N/P8	0 .55	0 .33	1 .00	1 .00	0 .23	0 .78	0 .63	0 .61	0 .64	0 .41	0 .58	1 .00	1 .00	0 .53	1 .00	1 .00	0 .00	

Statistical analysis of data presented in Figure 5a

1-way ANOVA, followed by Tukey post hoc.

Multivariate Tests of Significance (Fig5 statistics.sta)
Sigma-restricted parameterization
Effective hypothesis decomposition

Effect	Test	Value	F	Effect df	Error df	p
Intercept	Wilks	0 .465277	9 .768697	2	17	0 .001498
Peptide	Wilks	0 .227583	3 .105870	12	34	0 .004629

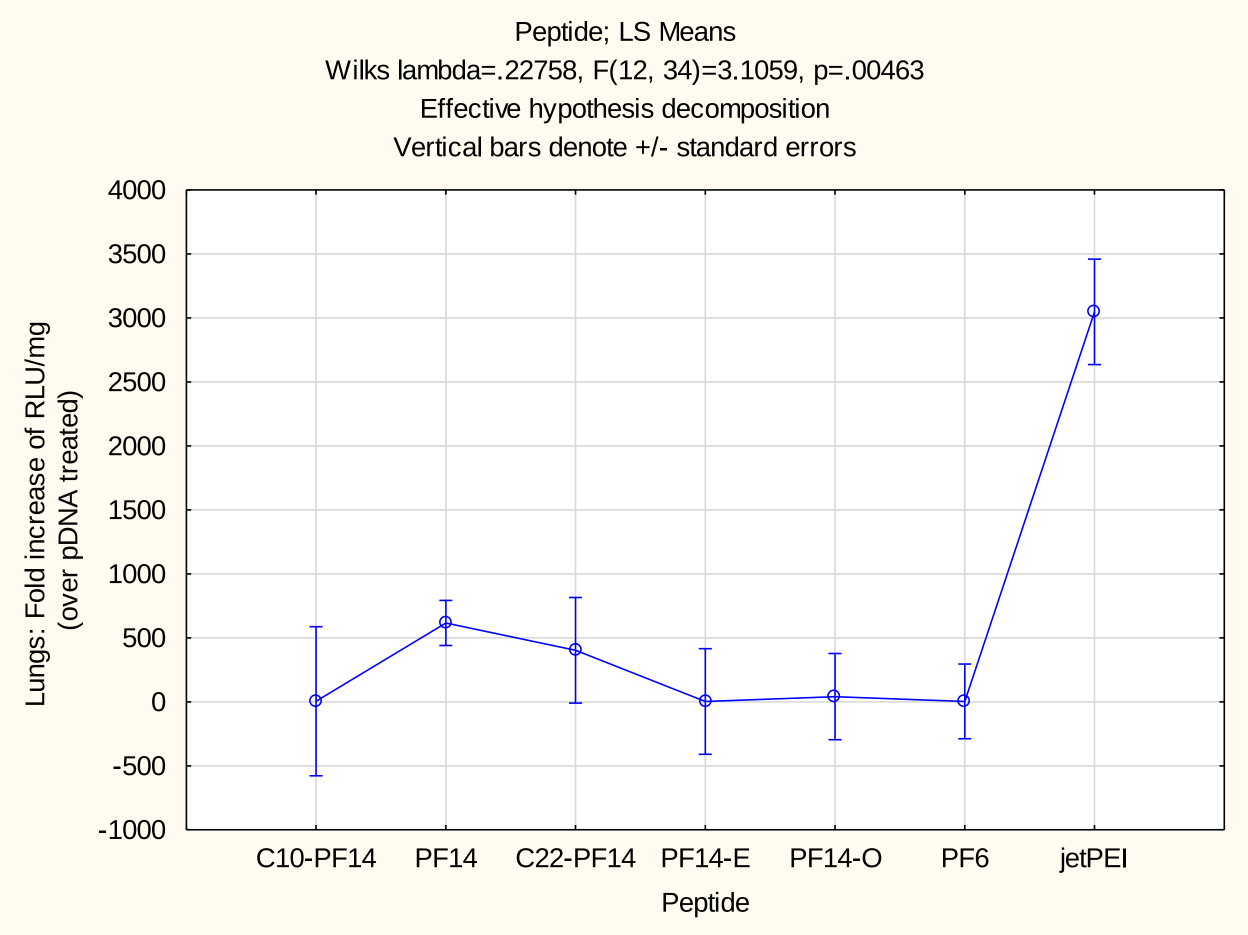

Tukey HSD test; variable Lungs (Fig5 statistics.sta)
Approximate Probabilities for Post Hoc Tests
Error: Between MS = 3398E2, df = 18.000

Cell No.	Peptide	{1} 4.8711	{2} 616.48	{3} 402.90	{4} 3.0412	{5} 41.018	{6} 3.5359	{7} 3047.4
1	C10-PF14		0 .95	1 .00	1 .00	1 .00	1 .00	0 .01
2	PF14	0 .95		1 .00	0 .81	0 .73	0 .56	0 .00
3	C22-PF14	1 .00	1 .00		0 .99	0 .99	0 .98	0 .00
4	PF14-E	1 .00	0 .81	0 .99		1 .00	1 .00	0 .00
5	PF14-O	1 .00	0 .73	0 .99	1 .00		1 .00	0 .00
6	PF6	1 .00	0 .56	0 .98	1 .00	1 .00		0 .00
7	jetPEI	0 .01	0 .00	0 .00	0 .00	0 .00	0 .00	

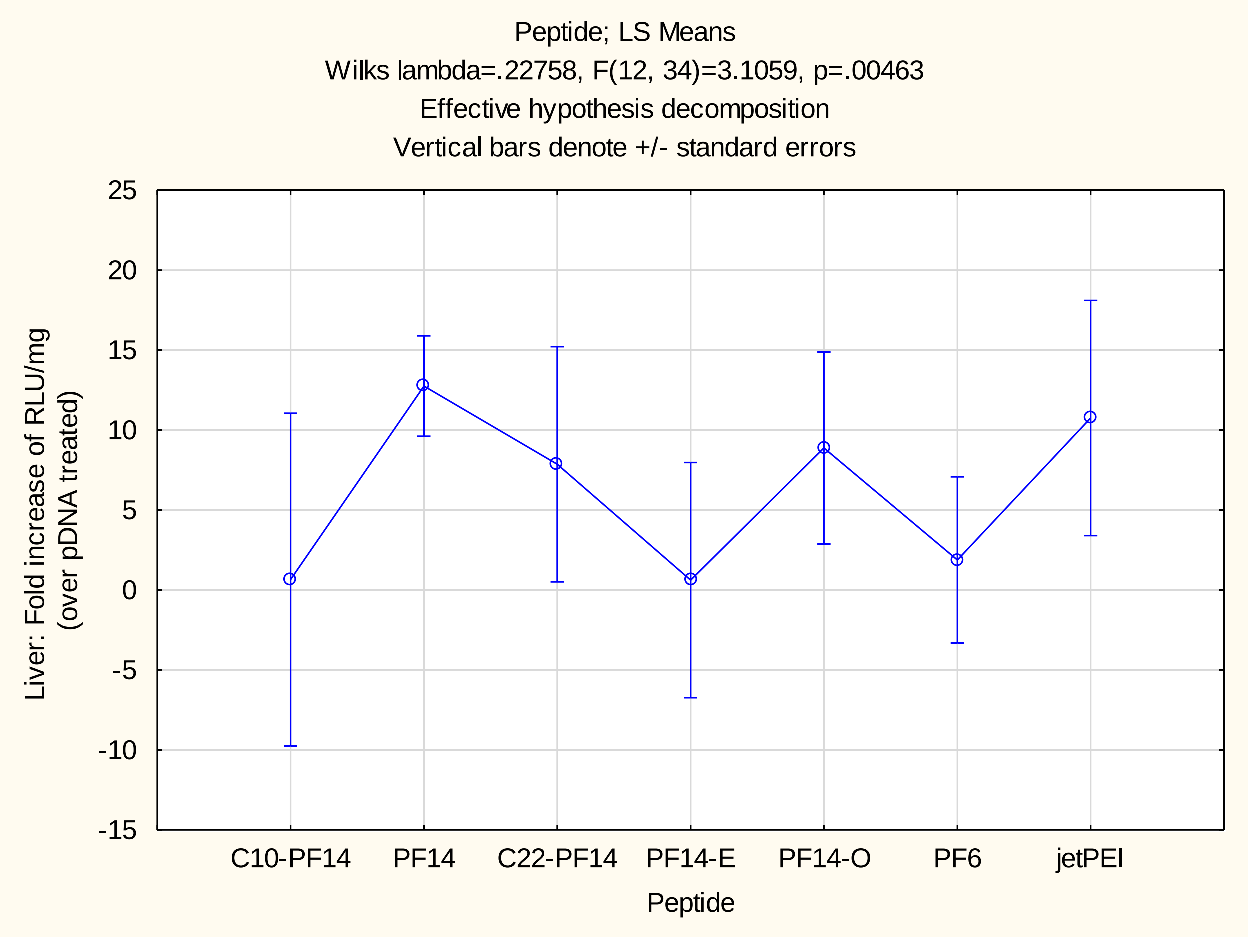

Tukey HSD test; variable Liver (Fig5 statistics.sta)
Approximate Probabilities for Post Hoc Tests
Error: Between MS = 108.15, df = 18.000

Cell No.	Peptide	{1} .65134	{2} 12.750	{3} 7.8621	{4} .61950	{5} 8.8769	{6} 1.8790	{7} 10.751
1	C10-PF14		0 .92	1 .00	1 .00	0 .99	1 .00	0 .98
2	PF14	0 .92		1 .00	0 .73	1 .00	0 .57	1 .00
3	C22-PF14	1 .00	1 .00		0 .99	1 .00	0 .99	1 .00
4	PF14-E	1 .00	0 .73	0 .99		0 .97	1 .00	0 .95
5	PF14-O	0 .99	1 .00	1 .00	0 .97		0 .97	1 .00
6	PF6	1 .00	0 .57	0 .99	1 .00	0 .97		0 .95
7	jetPEI	0 .98	1 .00	1 .00	0 .95	1 .00	0 .95	

Statistical analysis of data presented in Figure 5b

1-way ANOVA, followed by Tukey post hoc.

Effect	Multivariate Tests of Significance (Fig5 statistics.sta)<br>Sigma-restricted parameterization<br>Effective hypothesis decomposition<br>Test	Value	F	Effect df	Error df	p
Intercept	Wilks	0 .473000	6 .127898	2	11	0 .016283
Peptide	Wilks	0 .275588	3 .317935	6	22	0 .017624

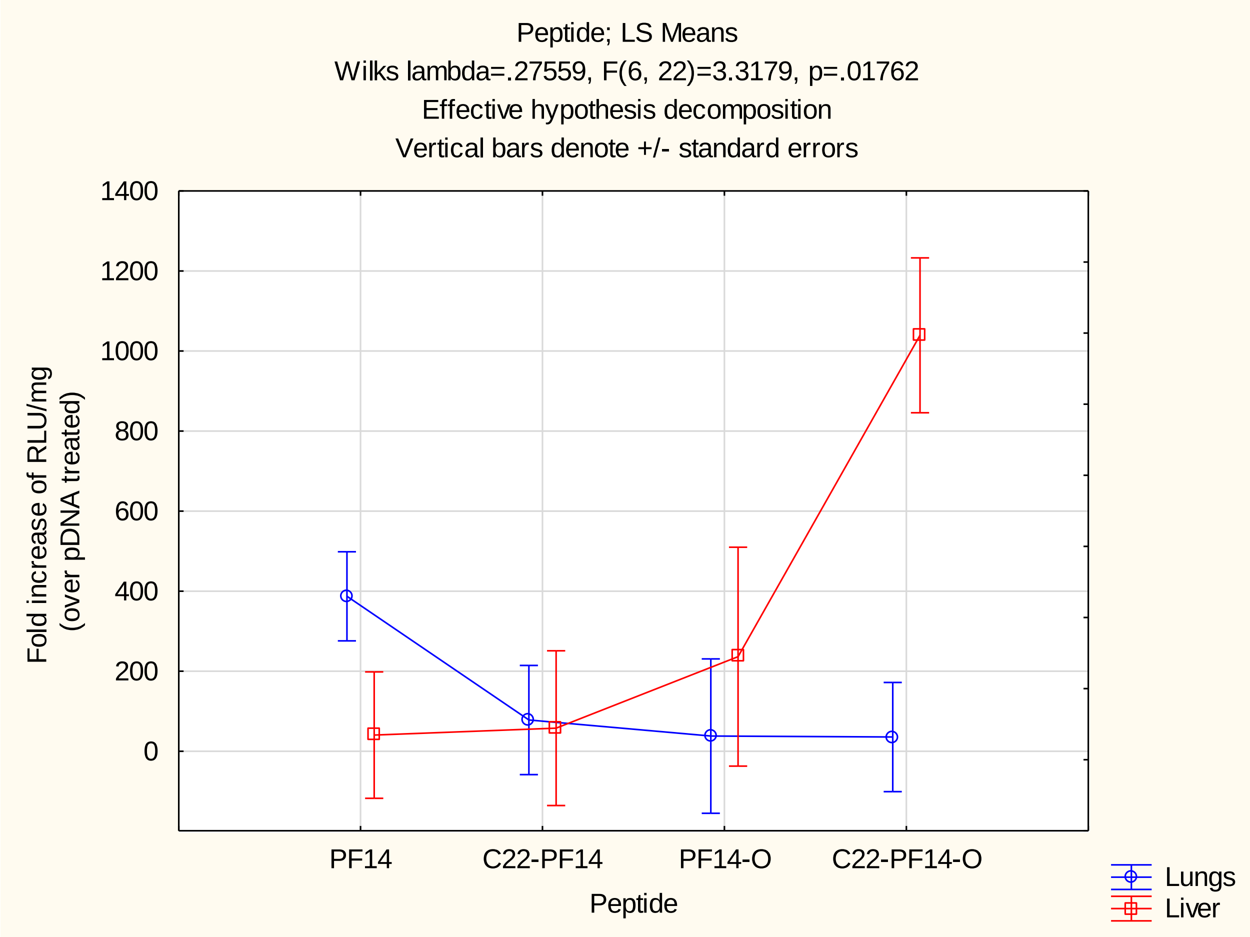

Cell No.	Tukey HSD test; variable Lungs (Fig5 statistics.sta)<br>Approximate Probabilities for Post Hoc Tests<br>Error: Between MS = 74420., df = 12.000<br>Peptide	{1}<br>387.13	{2}<br>77.968	{3}<br>37.898	{4}<br>35.571
1	PF14		0 .34	0 .43	0 .24
2	C22-PF14	0 .34		1 .00	1 .00
3	PF14-O	0 .43	1 .00		1 .00
4	C22-PF14-O	0 .24	1 .00	1 .00	

Cell No.	Tukey HSD test; variable Liver (Fig5 statistics.sta)<br>Approximate Probabilities for Post Hoc Tests<br>Error: Between MS = 1496E2, df = 12.000<br><br>Peptide	{1}<br>40.341	{2}<br>57.586	{3}<br>236.34	{4}<br>1039.3
1	PF14		1 .00	0 .92	0 .01
2	C22-PF14	1 .00		0 .95	0 .02
3	PF14-O	0 .92	0 .95		0 .13
4	C22-PF14-O	0 .01	0 .02	0 .13	
